# Zinc‐Doping‐Induced Electronic States Modulation of Molybdenum Carbide: Expediting Rate‐Determining Steps of Sulfur Conversion in Lithium‐Sulfur Batteries

**DOI:** 10.1002/advs.202417126

**Published:** 2025-03-31

**Authors:** Bin Qin, Yanmei Li, Qun Wang, Si Zhang, Jinglin Zhang, Bin Wang, Peijia Wang, Yuhan Chen, Weiqi Yao, Fang Wang

**Affiliations:** ^1^ Key Laboratory of Magnetic Molecules and Magnetic Information Materials of Ministry of Education & School of Chemistry and Materials Science Shanxi Normal University Taiyuan 030001 China; ^2^ Shanxi Key Laboratory of Advanced Magnetic Materials and Devices & Research Institute of Materials Science of Shanxi Normal University Taiyuan 030001 China; ^3^ Basic Department Shanxi Agricultural University Jinzhong 030801 China; ^4^ School of Materials Science and Engineering Harbin Institute of Technology Harbin 150001 China; ^5^ Department of Mechanical Engineering City University of Hong Kong Hong Kong SAR 999077 China; ^6^ Materials Science and Engineering Program & Texas Materials Institute The University of Texas at Austin Austin Texas 78712 USA

**Keywords:** catalytic host, d‐band center, lithium‐sulfur batteries, rate‐determining step, Zn‐doping

## Abstract

Enhancing Li_2_S deposition and oxidation kinetics in lithium‐sulfur batteries, especially the potential‐limiting step under lean electrolyte, can be effectively achieved by developing conductive catalysts. In this study, by using ZnMoO_4_ as precursors, Zn‐doped molybdenum carbide microflowers (Zn‐Mo_2_C) composed of speared porous sheets are fabricated with a hierarchically ordered structure. Density functional theory calculations indicate that Zn doping shifts the d‐band center on Mo atoms in Mo_2_C upward, promotes the elevation of certain antibonding orbitals in Mo─S bonds above the Fermi level, enhances d‐p interaction between lithium polysulfides (LiPSs) and catalysts, weakens both S─S and Li─S bonds of LiPSs. Incorporating Zn significantly reduces the Gibbs free energy barrier for the rate‐limiting step of the Li_2_S_2_ → Li_2_S conversion, from 0.52 eV for Mo_2_C to just 0.05 eV for Zn‐doped Mo_2_C. Thus, the synthesized Zn‐Mo_2_C demonstrates impressive bifunctional electrocatalytic performance, significantly advancing sulfur reduction and Li_2_S decomposition. Moreover, this modification enhances charge transfer within the Zn‐Mo_2_C/LiPSs system, synergistically accelerating the kinetics of Li_2_S_4_ to Li_2_S reduction and Li_2_S oxidation. The Zn‐Mo_2_C/S cathode demonstrates impressive electrochemical performance, achieves remarkable cycling stability with a minimal capacity decay of 0.021% per cycle over 1000 cycles at 5 C, underscoring its potential for high‐energy applications.

## Graphical abstract

Zn‐doped molybdenum carbide microflowers (Zn‐Mo_2_C) are exploited as a sulfur host for Li–S batteries. Zn doping elevates the d‐band center on Mo, enhancing LiPSs anchoring, weakening S─S/Li─S bonds, and reducing Li_2_S_2_→Li_2_S conversion energy barrier from 0.52 to 0.05 eV. Therefore, Zn‐Mo_2_C/S cathodes with accelerated sulfur redox kinetics and improved charge transfer achieve excellent sulfur electrochemistry.

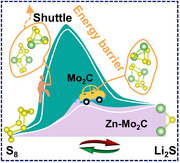

## 1 Introduction

Lithium‐sulfur batteries (LSBs), known for their high energy density, stand out as leading contenders for next‐generation energy storage.^[^ ^1^, ^2^, ^3^ ^]^ However, the deposition of Li_2_S, a crucial process accounting for 75% of the overall capacity, poses significant challenges due to its slow kinetics. Li_2_S, the terminal reduction product of S_8_, exhibits inferior conductivity (σ: 1 × 10^−9^ S cm⁻¹) and inherently sluggish kinetics in both its deposition and oxidation processes, posing significant challenges to efficient operation. As the ultimate reduced product of S_8_, Li_2_S suffers from insulating characteristics (σ: 1 × 10^−9^ S cm^−1^) and inherently slow deposition and oxidation kinetics, which hinder its electrochemical performance.^[^ ^4^ ^]^ The critical kinetic challenge arises from the substantial interfacial impedances caused by the ionic and electronic insulator discharge product, Li_2_S. The initial nucleation of Li_2_S encounters significant resistance at the heterogeneous interface between the electrolyte and the cathode, creating a distinct overpotential at the onset of deposition.^[^ ^5^ ^]^ The Gibbs free energies of the Li_2_S_2_ → Li_2_S (ΔG = 0.6–1.4 eV) are nearly four times that of others such as from Li_2_S_8_ to Li_2_S_4_ (ΔG = 0.01–0.4 eV).^[^ ^6^ ^]^

Recent advancements have seen the use of dual‐function conductive and catalytic materials as additives in sulfur cathodes.^[^ ^7^, ^8^, ^9^ ^]^ These materials expedite polysulfide conversion and modulate the kinetics of the associated reactions. Transition metal carbides (TMCs), like Mo_x_C, have recently emerged as promising sulfur host materials for lithium‐sulfur battery cathodes, a conclusion supported by our work and other recent studies. These materials exhibit significant advantages, including strong polarity, exceptional catalytic efficiency, and excellent electrical conductivity (e.g., Mo_2_C: 1.41 × 10^4^ S cm^−1^), positioning them as promising sulfur hosts in LSB cathodes.^[^ ^10^, ^11^, ^12^, ^13^ ^]^ However, high‐temperature annealing in traditional TMC synthesis typically causes particle sintering and aggregation, adversely affecting morphology control and reducing specific surface area, which together impairs the catalytic activity.

Recent findings establish d‐band center modulation as a potent strategy for engineering the electronic structures of catalyst crystals and refining their interactions with the frontier orbitals of polysulfides, which show that doping or inducing lattice strain can enhance catalytic activity by shifting the d‐band centers of electrocatalysts.^[^ ^14^, ^15^ ^]^ Elemental doping offers a viable strategy to enhance the adsorption properties and catalytic performance of Mo_x_C. By fine‐tuning the atomic configuration and electronic structure, this approach not only improves internal conductivity but also introduces additional active sites for catalytic processes.^[^ ^14^, ^15^, ^16^, ^17^, ^18^, ^19^ ^]^ For example, Yang's team pioneered the use of zinc single‐atom‐doped Ti_3_C_2_ MXenes (SA‐Zn‐MXenes) as sulfur hosts. These materials not only demonstrated robust interactions with polysulfides but also effectively catalyzed their conversion by lowering the energy barrier for the Li_2_S_4_ to Li_2_S_2_/Li_2_S transition from 0.92 to 0.71 eV. The electronic structure of MXene can be significantly modulated by Zn atoms.^[^ ^6^ ^]^ Xu et al. have developed a Zn‐doped CoTe_2_ composite, where the Jahn–Teller effect facilitated by low Zn^2+^ doping results in mild lattice strain and increased exposure of active sites in Co_0.9_Zn_0.1_Te_2_. This improvement in catalytic activity accelerates the electrochemical kinetics of the sulfur cathode.^[^ ^20^ ^]^

Zn, a transition metal with a melting point of 419.53 °C and a boiling point of 907 °C, starts to volatilize at temperatures above 650 °C. By the low boiling point, Zn gradually escapes via volatilization, facilitating the synthesis of single‐metal atoms,^[^ ^21^ ^]^ yolk‐shell structures,^[^ ^22^ ^]^ or Zn‐doped electrocatalysts.^[^ ^23^ ^]^ Herein, inspired by the above discoveries, by using ZnMoO_4_ as the precursor, a simple two‐step approach was developed to synthesize Zn‐doped porous Mo_2_C micron‐scale flowers, featuring an architecture of interconnected speared nanosheets (denoted as Zn‐Mo_2_C). Zn‐Mo_2_C, featuring a hierarchically integrated conductive network with multiscale pores, delivers extensive active sites and high‐speed electron/ion pathways, enabling efficient trapping and conversion of polysulfides. Electrochemical evaluations combined with density functional theory (DFT) analyses revealed that the doping of Zn significantly enhances the interaction between Mo_2_C and LiPSs, effectively mitigating the shuttle effect. Furthermore, the doping of Zn sites weakens the interatomic bonds of LiPSs, facilitates the charge transfer in the Zn‐Mo_2_C/LiPSs system, and thus greatly improves the LiPSs conversion kinetics. By utilizing Zn‐Mo_2_C as the sulfur host, the cathode demonstrates a remarkable capacity of 849 mAh g^−1^ at 2 C, alongside exceptional cycling stability, maintaining a low capacity fading of 0.025% per cycle over 500 cycles.

## 2 Result and Discussion

**Figure 1:**
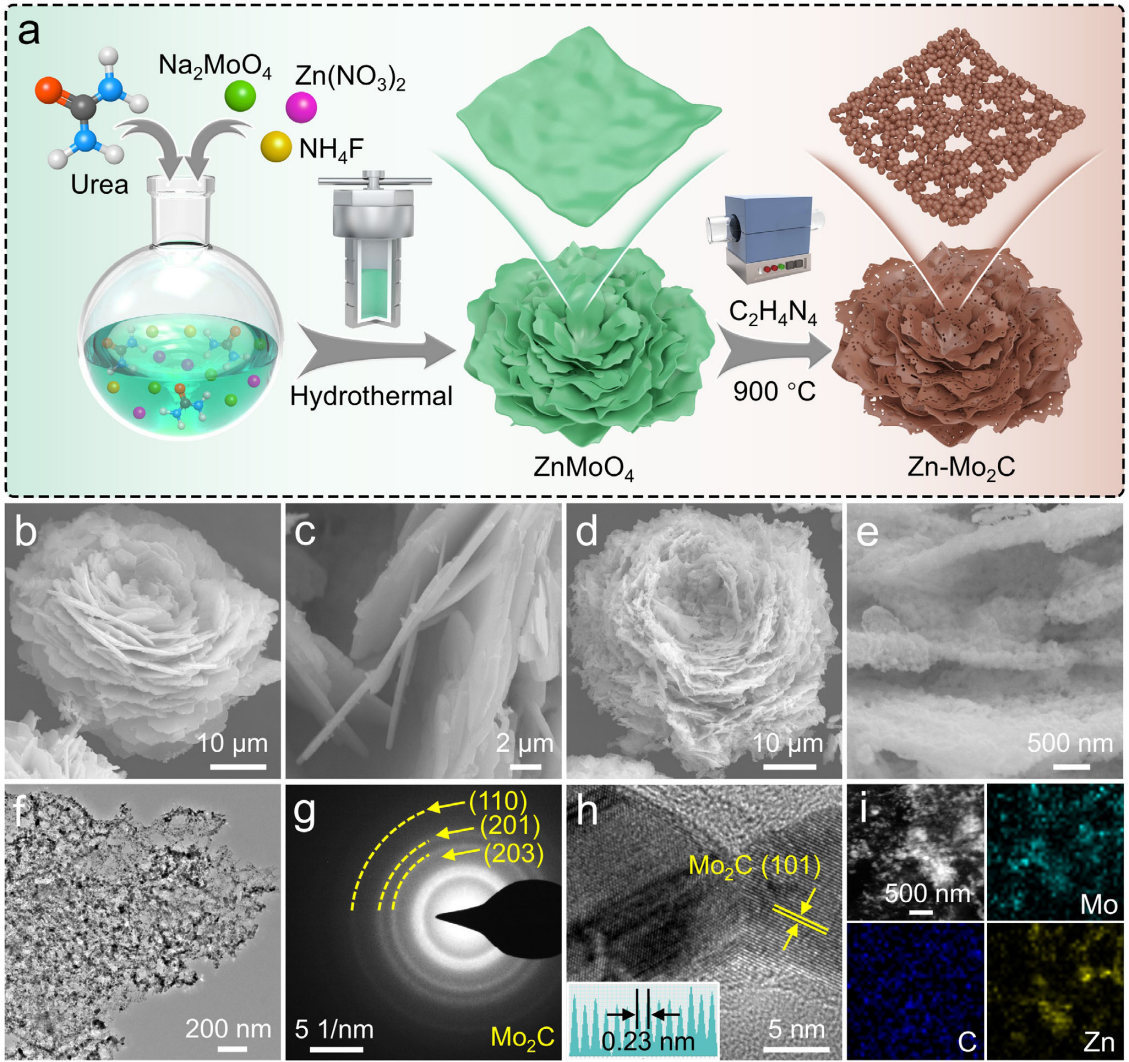
a) Schematics illustrating the synthesis process of Zn‐Mo_2_C. b,c) FESEM images of ZnMoO_4_. d,e) FESEM of Zn‐Mo_2_C, f) TEM image of Zn‐Mo_2_C, g) the SAED pattern of Zn‐Mo_2_C. h) HRTEM image of Zn‐Mo_2_C. i) HAADF‐STEM and the corresponding element mapping images of Zn‐Mo_2_C.

The Zn‐Mo_2_C was prepared by hydrothermal reaction and subsequent annealing treatment, and the corresponding synthetic procedures are illustrated in **Figures** **1**a and S1 (Supporting Information). Specifically, after a facile solvothermal reaction, SEM images of ZnMoO_4_ show uniform flower‐like morphology, composed of speared sheets (Figure 1b,c; Figure S2, Supporting Information). The majority of the synthesized corrugated sheets demonstrate a consistent thickness of approximately 300–400 nm (Figure 1c). Then, in the presence of dicyandiamide, through the one‐step pyrolysis reaction of ZnMoO_4_, the Zn‐Mo_2_C micron flowers can be in situ formed. The resulting Zn‐Mo_2_C micron flowers maintain the original morphology except that the surface becomes rough (Figure 1d,e). Particularly, as shown in Figure S3 (Supporting Information), peaks of Zn‐Mo_2_C are indexed as hexagonal Mo_2_C (PDF#35‐0787). Remarkably, no shift in the XRD peaks was observed upon doping a small amount of Zn atoms, no shift of the XRD peak can be observed, which may be attributed to the similar atomic radius of Zn (1.39 Å) to that of Mo (1.40 Å). Each petals are composed of homogeneously dispersed nanoparticles (Figure 1e,f), and the polycrystalline structure is corroborated by the clear diffraction rings in the selected area electron diffraction (SAED) pattern (Figure 1g). High‐resolution transmission electron microscopy (HRTEM) images (Figure 1h) reveal an interplanar spacing of 0.23 nm, which matches the (101) facets of Mo_2_C, which is in accordance with the XRD pattern of Zn‐Mo_2_C depicted in Figure S3 (Supporting Information). HAADF STEM and the elemental distribution maps demonstrate that Zn, Mo, and C elements are homogeneously distributed (Figure 1i). The content of Zn in Zn‐Mo_2_C was measured by inductively coupled plasma‐optical emission spectroscopy (ICP‐OES), which was determined to be 2.1 wt.%.

**Figure 2:**
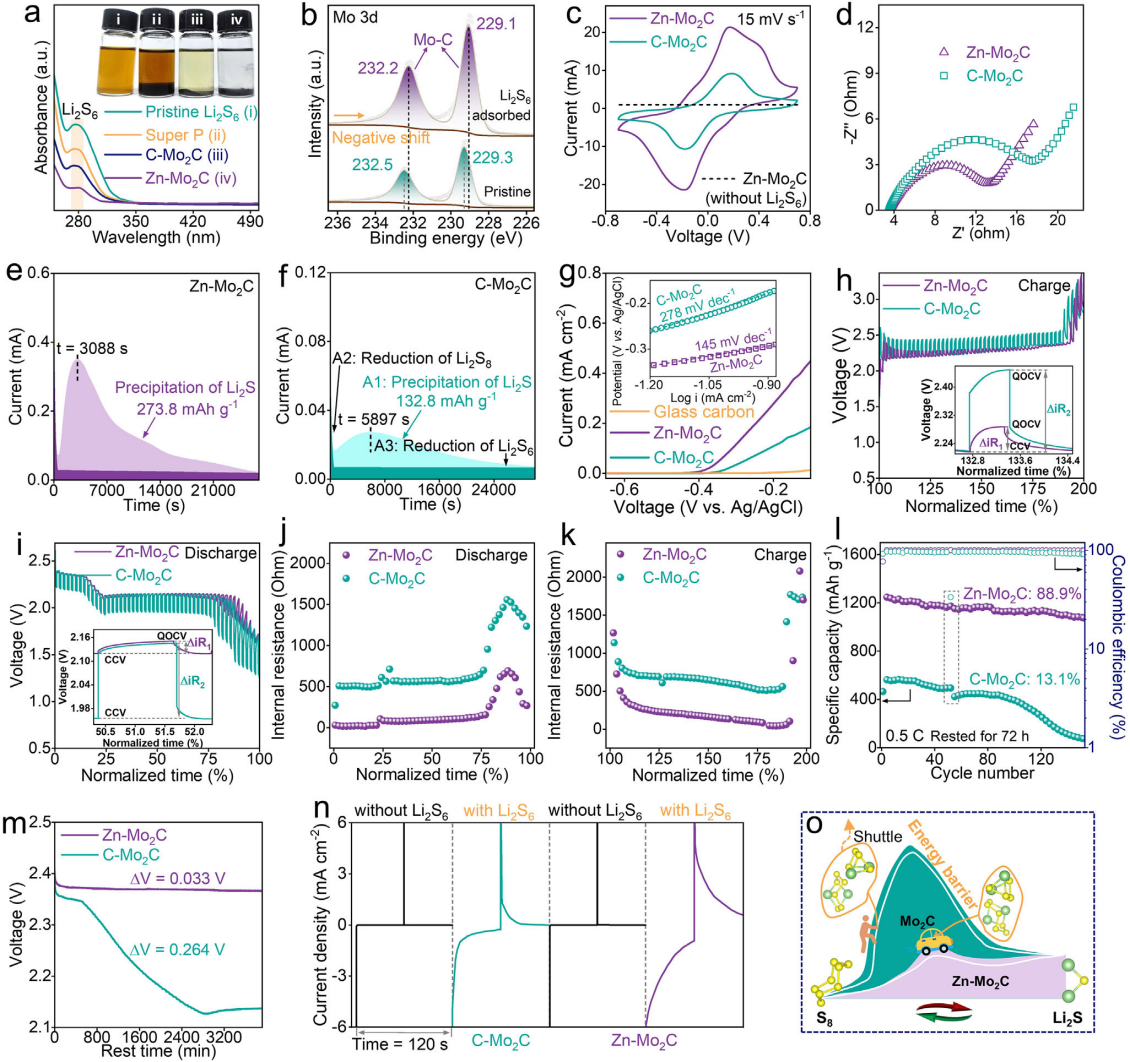
a) LiPSs adsorption 2a, inset shows the photograph of different samples soaked in Li_2_S_6_ solution for 12 h. b) Mo 3d high‐resolution XPS spectra of Zn‐Mo_2_C/ Li_2_S_6_. c) CV curves and d) EIS spectra of symmetric cells. Potentiostatic nucleation profiles of Li_2_S_8_ solution on the e) Zn‐Mo_2_C and f) C‐Mo_2_C electrodes. g) LSV curves, inset shows the corresponding Tafel plots. GITT profiles of sulfur cathodes during h) charge and i) discharge. j,k) The corresponding internal resistances of the batteries *vs*. normalized charge/discharge time. l) Self‐discharge cycling performance at 0.5 C. m) Open circuit voltage profiles during the self‐discharge test. n) Chronoamperometry curves. o) Schematic illustration of catalytic conversion of LiPSs by the Mo_2_C and Zn‐Mo_2_C host during the charge/discharge processes.

To explore the adsorption capabilities of these samples toward LiPSs, equal surface areas of Zn‐Mo_2_C, C‐Mo_2_C, and Super P were immersed into the Li_2_S_6_ solution. As depicted in the inset of **Figure** **2**a, after adsorption for 2 h, the solution containing Zn‐Mo_2_C is nearly transparent. In comparison, the Li_2_S_6_ solution with C‐Mo_2_C stayed yellow, highlighting the enhanced polysulfide adsorption capacity due to Zn doping. Additionally, the UV–vis absorption spectra of the supernatants were examined (Figure 2a), showing a decreasing trend in polysulfide adsorption capacity as follows: Zn‐Mo_2_C > C‐Mo_2_C > Super P. Significantly, the polysulfide signal for the Zn‐Mo_2_C sample was barely detectable, indicating its superior adsorption capacity compared to C‐Mo_2_C. The interactions between catalysts and Li_2_S_6_ were further examined using XPS analysis. The Mo 3d signals of Zn‐Mo_2_C shift to higher binding energies upon polysulfide adsorption, indicating a transfer of electrons from Mo to the polysulfide species.

**Figure 3:**
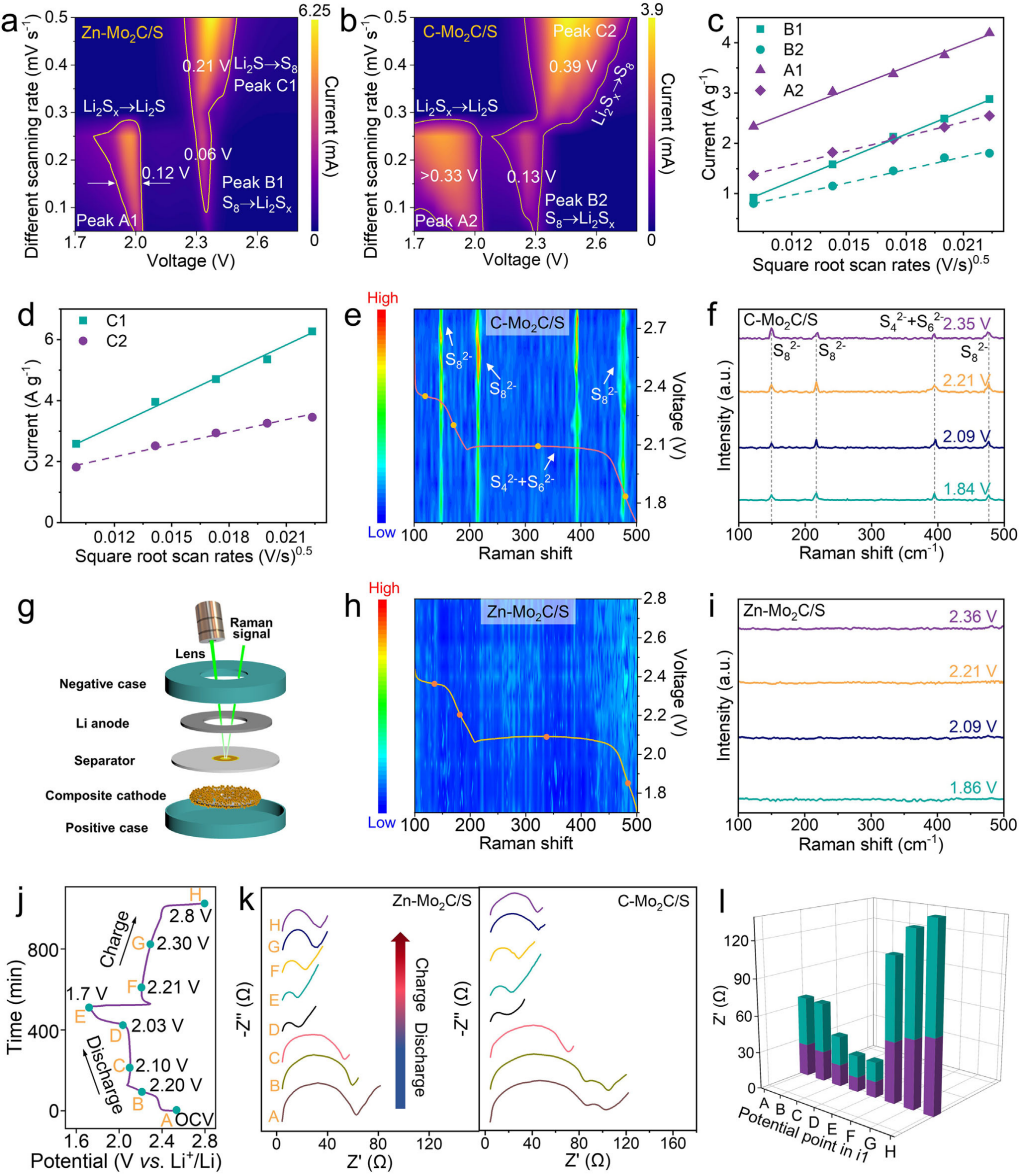
Contour plots of CV curves for a) Zn‐Mo_2_C and b) C‐Mo_2_C with different scan rates. c,d) Peak currents versus square root of scan rates of Zn‐Mo_2_C (A1, B1, C1) and C‐Mo_2_C (A2, B2, C2). e) Contour images of in situ Raman spectra for Li−S batteries with e) Zn‐Mo_2_C and h) C‐Mo_2_C electrode during discharging at 0.1 C. In situ Raman spectra of the f) Zn‐Mo_2_C and i) C‐Mo_2_C electrode. g) Schematic illustration of in situ Raman detection. The j) charge/discharge profiles and k) ex situ EIS spectra for the Zn‐Mo_2_C cell at various states of charge and discharge. l) The corresponding *R*_ct_ histograms of Zn‐Mo_2_C (purple) and C‐Mo_2_C (cyan).

To further study the electrocatalytic activity of Zn‐Mo_2_C and C‐Mo_2_C, symmetric batteries were constructed using 0.2 M Li_2_S_6_ electrolyte and samples mounted on carbon paper (CP) as electrodes. As depicted in Figure 2c, CV curves were recorded over a voltage range of −0.7–0.7 V with Zn‐Mo_2_C and C‐Mo_2_C electrodes. The CV curves in Figure 2c show that the cell with Zn‐Mo_2_C electrodes achieves higher currents compared to the C‐Mo_2_C electrodes, suggesting that Zn doping significantly enhances the redox kinetics for the liquid‐liquid conversion of LiPSs. In addition, the EIS of the Zn‐Mo_2_C‐based symmetric cell shows the lowest charge‐transfer impedance (Figure 2d), indicating a reduced charge‐transfer barrier at the Zn‐Mo_2_C/LiPS interface, which facilitates the rapid conversion of LiPSs. Typically, uncontrolled nucleation and growth of electron‐ and ion‐insulating Li_2_S on the cathode surface disrupt the continuous conversion of LiPSs, significantly hampering the kinetics of the reaction. In this regard, a Li_2_S precipitation experiment was conducted to reveal the efficient catalytic conversion of LiPSs facilitated by Zn‐Mo_2_C. The capacities of the Li_2_S deposit on various substrates were calculated according to Faraday's law.^[^ ^24^, ^25^ ^]^ As shown in Figure 2e, the area capacity of Zn‐Mo_2_C reaches 273.8 mAh g^−1^ with a rapid current response of 3.09 × 10^3^ s. Comparatively, C‐Mo_2_C endows lower area capacities of 132.8 mAh g^−1^ and slower current responses (5.9 × 10^3^ s, **Figure** **3**f). Zn‐Mo_2_C, characterized by faster Li_2_S nucleation/growth and higher deposition capacity, underscores the following points: i) Compared to C‐Mo_2_C, Zn‐Mo_2_C facilitates faster catalytic conversion kinetics between LiPSs and Li_2_S due to the doping of Zn; ii) The rapid current response of Zn‐Mo_2_C shows quick Li_2_S growth; iii) The higher Li_2_S deposition capacity of Zn‐Mo_2_C suggests a more efficient conversion of LiPSs to Li_2_S.

The catalytic oxidation behavior of Li_2_S on these samples was evaluated using three‐electrode linear sweep voltammetry (LSV). As shown in Figure 2g, it can be noted that the Zn‐Mo_2_C electrode delivers an onset potential of −0.42 V for the Li_2_S oxidization, which is much lower than that of C‐Mo_2_C (−0.36 V), indicating the lower energy barrier to initiate the Li_2_S decomposition. Moreover, the Zn‐Mo_2_C electrode also demonstrates a higher current response among these electrodes, corresponding to the considerably reduced energy barrier and enhanced reaction kinetics with the doping of Zn. This catalyzed reaction behavior can also be confirmed by the smaller Tafel slope of 145 mV dec^−1^ for the Zn‐Mo_2_C electrode compared with 278 mV dec^−1^ for C‐Mo_2_C in the according to Tafel plot (inset of Figure 2g).

Galvanostatic intermittent titration measurements (GITT) are carried out to further explore the internal resistances of LSBs. As shown in Figure 2h,i, a typical charge−discharge voltage platform is observed, and the internal reaction resistances (ΔR_internal_) at different discharge/charge stages were calculated according to the following equation:^[^ ^26^, ^27^ ^]^

(1) $$\Delta R_{\mathrm{internal}}\left(\Omega \right)=\left|\Delta V_{\mathrm{QOCV}-\mathrm{CCV}}\right|/I_{\mathrm{applied}}$$

Here, ΔV indicates the voltage difference between the closed and quasi‐open circuit points, with I_applied_ representing the applied current. Figure 2j,k clearly shows that the Zn‐Mo_2_C cell maintains a much lower internal resistance throughout the lithiation and delithiation processes compared to C‐Mo_2_C. This significant reduction underscores the role of Mo_2_C in boosting the reaction kinetics within the battery system. Distinctly, the ΔR_internal_ in the nucleation process is significantly lower than that in the dissolution and transformation process, indicating that the energy barrier needed to be overcome in the dissociation stage is higher.^[^ ^28^ ^]^ Li‐S batteries are hindered by a critical self‐discharge issue during storage, stemming from the shuttling of LiPSs across the separator to the lithium anode, which poses a major drawback for their practical use. The severe self‐discharge induced by this shuttle effect was investigated by tracking the open circuit voltages (OCVs) at 0.5 C. After 52 cycles and a full charge of 2.8 V, the batteries were rested for 72 h before undergoing an additional 100 charge‐discharge cycles. C‐Mo_2_C cells showed notable self‐discharge, with a 41.3% capacity loss post‐rest (Figure S5b,d, Supporting Information). Notably, the first discharge plateau at ≈2.3 V almost vanished, attributed to the conversion of high‐order LiPSs to lower‐order ones via internal shuttling. Conversely, Zn‐Mo_2_C cells maintained 95.9% capacity after 72 h (Figure S5a,c, Supporting Information), highlighting its effectiveness in mitigating the shuttling effect of LiPSs, as evidenced by the robust cycling performance (Figure 2l) and the well‐maintained high discharge plateau during rest test (Figure 2m).

The chronoamperometry data reveal that cells containing Li_2_S_6_ exhibit a markedly higher current response than those without Li_2_S_6_ (Figure 2n). This finding implies that the lithiation/delithiation reactions, rather than double‐layer capacitance, are responsible for the current observed. Simultaneously, the Zn‐Mo_2_C symmetric cell demonstrates a higher current response over C‐Mo_2_C, highlighting its substantial role in enhancing the conversion kinetics of polysulfides. Based on the above electrochemical tests, the Zn‐Mo_2_C material showcases stronger LiPSs affinity and better catalytic activities in comparison to C‐Mo_2_C, which eventually enhances the utilization rate and reaction kinetics of active sulfur, refers to the schematic diagram in Figure 2O. Figure S18a,c (Supporting Information) depicts the morphology of the lithium metal anode after cycling, where Zn‐Mo_2_C exhibits a significantly smoother and more uniform surface than C‐Mo_2_C, in agreement with the SEM analysis in Figure S18e,f (Supporting Information). Figure S18b,d (Supporting Information) reveals that polysulfide species from the C‐Mo_2_C cathode migrate more readily across the PP separator, leading to pronounced sulfur deposition on the anode, as further confirmed by EDS mapping (Figure S18g,h, Supporting Information). Notably, the sulfur content on the cycled Zn‐Mo_2_C anode is only 0.9 wt.%, significantly lower than the 11.0 wt.% observed for C‐Mo_2_C.

Cyclic voltammetry (CV) tests of the coin cells were performed at a sweep rate of 0.1–0.5 mV s^−1^ to investigate the enhanced reaction kinetics, as shown in Figure S6 (Supporting Information). **Figure** **3**a,b display the contour plots of the CV curves for Zn‐Mo_2_C and C‐Mo_2_C, respectively. Each cell exhibits two cathodic peaks and a single anodic peak. The cathodic peak at 2.3 V (Peak B) corresponds to the reduction of sulfur to long‐chain Li_2_S_x_ (4 ≤ x ≤ 8), whereas the peak at 2.0 V (Peak A) is associated with the formation of the insoluble short‐chain products Li_2_S_2_/Li_2_S. In the oxidation process, the anodic scan reveals a shift from lithium sulfides (Li_2_S_2_/Li_2_S) to lithium polysulfides (LiPS) and sulfur, marked by an overlapping peak in the 2.4–2.6 V range (Peak C). Importantly, as shown in Figure S7 (Supporting Information), compared with C‐Mo_2_C based cell, the Zn‐Mo_2_C based cell delivers lower polarization voltages (Figure S7b, Supporting Information), higher current response (Figure S7c, Supporting Information), and narrower half‐peak width (Figure 3a,b) at various sweep rates, indicating the enhanced redox reactions kinetics toward LiPS conversion of Zn‐Mo_2_C. As depicted in Figure 3c,d, the cathodic peak (C1 and C2) and anodic peak (A) currents of the cells exhibit linear relationships with the square root of scan rates, signifying its diffusion‐limited characterization. To investigate the lithium‐ion diffusion kinetics, the lithium‐ion diffusion coefficient ($D_{Li^{+}}$) was estimated by using the Randles–Sevcik equation:^[^ ^29^, ^30^ ^]^

(2) $$I_{p}=\left(2.65\times 10^{5}\right)n^{1.5}A{D_{\mathrm{Li}}}^{0.5}C_{\mathrm{Li}}v^{0.5}$$

where *I*_p_ is peak current (A), n is the number of electrons transferred in the reaction (for Li‐S battery, *n* = 2), *A* is the area of the electrode (1.13 cm^2^), *D*_Li_ is lithium‐ion diffusion coefficient (cm^2^ s^−1^), *C*_Li_ is concentration of Li^+^ in the electrolyte (1 × 10^−3^ mol cm^−3^), and *v* is sweeping rate (V s^−1^). As summarized in Table S1 (Supporting Information), the *D*_Li_ values of Zn‐Mo_2_C cell are 3.05 × 10^−7^, 4.70 × 10^−8^, and 3.72 × 10^−7^ cm^2^ s^−1^ for cathodic (A, B) and anodic peak (C), respectively, higher than the reference values of C‐Mo_2_C. Such accelerated Li ion transport could play critical roles in enhancing the performance of Li–S batteries. In situ Raman spectroscopy was executed to gain insight into the effectiveness of mitigating LiPS shuttle. During the discharge processes of Zn‐Mo_2_C containing cathodes, only a few signals of S_8_^2−^ were detected, demonstrating effective shuttle effect suppression (Figure 3h,i). Conversely, cathodes with C‐Mo_2_C exhibit prominent peaks at 149.3, 217.1, 394.9, and 281.5 cm^−1^, associated with S_8_^2−^, S_6_^2−^, and S_4_^2−^, throughout the discharge (Figure 3e,f), indicating a pronounced shuttle effect irreversible depletion of LiPSs. In addition, the charge transfer characteristics of the Zn‐Mo_2_C/S cathode were investigated using in situ electrochemical impedance spectroscopy (EIS) during the first cycle of charging and discharging (Figure 3j). The two depressed semicircles observed in the high‐ and middle‐high‐frequency regions are associated with interfacial resistance and charge transfer resistance, respectively. During discharging, a slight decrease in *R*_ct_ was observed, attributed to the dissolution of sulfur. Upon recharging, the interface resistance and charge transfer resistance remained largely unchanged, implying a stable interface reaction. Impressively, the *R*_ct_ of the Zn‐Mo_2_C/S cathode remained consistently lower than that of the C‐Mo_2_C/S cathode throughout the discharge/charge cycle (Figure 3l), confirming rapid electron transfer within the 3D continuous framework of Zn‐Mo_2_C/S. At the end of the recharge (Figure 3k), the charge transfer resistance and interface resistance are reduced compared to the original state, suggesting that the Zn‐Mo_2_C catalyst could optimize sulfur deposition. These observations demonstrate that the Zn‐Mo_2_C host exhibits enhanced conductivity, rapid Li‐ion transfer, and improved redox conversion kinetics, aligning with the outstanding rate performance of the corresponding Li‐S cell.

**Figure 4:**
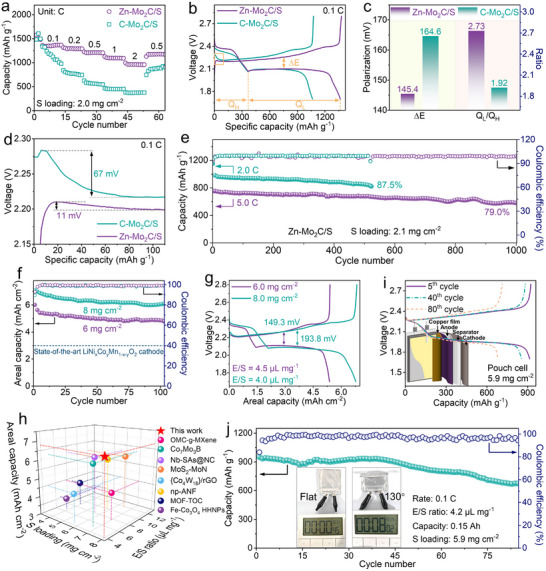
Electrochemical performances of the Mo_2_C and Zn‐Mo_2_C based Li‐S batteries. a) Rate performance. b) GCD profiles at 0.1 C. c) The value of ΔE and Q_L_/Q_H_ obtained from charge−discharge curves of various electrodes. d) Initial charge voltage profiles. e) Cycling performance. f) Cycling performance of the high‐sulfur‐loading electrodes, and g) charge–discharge profiles at 0.2 C. h) Comparisons of 3D plots for high‐sulfur loading Li‐S batteries. i) GCD profiles of the Li‐S pouch cell, the inset illustrates the schematic of the pouch cell. j) Cycling performance at 0.1 C with for Li‐S pouch cell, the inset presents a schematic depiction of the pouch cell under different bending conditions, demonstrating its ability to consistently power an electronic timer.

To examine the effect of Zn doping on the adsorption and catalytic activity of the samples, we use commercial Mo_2_C particles (abbreviated as C‐Mo_2_C) as the control sample, the morphology and phase are shown in Figure S11 (Supporting Information). To evaluate the reaction kinetics in Li–S chemistry, we compared the rate capabilities of Zn‐Mo_2_C/S and C‐Mo_2_C/S cathodes, with a sulfur loading of ≈2.0 mg cm^−2^ and sulfur content of 67.4 wt.%. Figure S12a (Supporting Information) presents the thermogravimetric (TG) analysis of Zn‐Mo_2_C/S, showing that the conventional Zn‐Mo_2_C/S composite material (≈2.0 mg cm^−2^) contains approximately 67.4 wt.% sulfur. The composite was synthesized using a conventional thermal‐diffusion approach and then used as sulfur cathodes in 2032 coin cells. It is worth noting that the S contents in high‐sulfur‐loading electrodes (6.0 and 8.0 mg cm^−2^) are ≈79.6 wt.%, as depicted in Figure S12b (Supporting Information). As shown in **Figures** **4**a and S13 (Supporting Information), the Zn‐Mo_2_C/S electrode demonstrates reversible discharge capacities of 1368, 1300, 1219, 1098, and 965 mAh g^−1^ under current densities of 0.1, 0.2, 0.5, 1, and 2 C, respectively, surpassing those of C‐Mo_2_C. Upon the current density going back to 0.5 C again, the discharge capacity recovered to 1178 mAh g^−1^, reaching 96.6% of its initial value, confirming excellent electrochemical reversibility and high sulfur utilization efficiency. Figure 4c illustrates the polarization potential (ΔE), which refers to the voltage gap between the second discharge and the charge plateaus shown in the GCD curves in Figure 4b. The Zn‐Mo_2_C/S cell displays a reduced polarization potential of 145.4 mV relative to C‐Mo_2_C/S (ΔE = 164.6 mV), attributed to the heightened electrocatalytic efficiency of Zn‐Mo_2_C in the conversion of LiPSs. Q_H_ and Q_L_ are the respective capacities of the high and low discharge plateaus. The ratio Q_L_/Q_H_ serves as a metric for assessing catalytic activity in the LiPS conversion.^[^ ^31^, ^32^ ^]^ Therefore, a larger Q_L_/Q_H_ signifies superior catalytic capability. As illustrated in Figure 4b,c, in alignment with the aforementioned results, the Q_L_/Q_H_ values for Zn‐Mo_2_C/S electrodes (2.73) are higher than those for C‐Mo_2_C/S electrodes (1.92), further confirming that Zn‐Mo_2_C could enhance the reduction kinetics in Li‐S batteries. Examining the galvanostatic curves in detail, Figure 4d highlights the voltage rise at the initial charging period, corresponding to the overpotential necessary for Li_2_S activation. Notably, Zn‐Mo_2_C/S electrodes exhibited a markedly lower overpotential (11 mV) over C‐Mo_2_C/S electrodes (67 mV), confirming the enhanced activation process of Li_2_S facilitated by Zn‐Mo_2_C.

Ensuring long‐term cycling stability with high‐capacity retention is vital for the practical implementation of Li−S batteries. Accordingly, we examined the long‐cycling stability of the Zn‐Mo_2_C/S cells at rates of 2 C and 5 C, respectively (Figure 4e). Following 500 cycles at 2 C, the Zn‐Mo_2_C/S cathode sustained a discharge capacity of 849 mAh g^−1^, corresponding to an average capacity decay of 0.025% per cycle. To substantiate the Zn‐Mo_2_C/S cathode's rate performance and stability, testing was conducted at 5 C. The battery delivered an initial discharge capacity of 740 mAh g^−1^, with a decay rate of 0.021%. Importantly, the two distinct discharge plateaus are well maintained after extended cycling tests (Figure S14, Supporting Information). It is worth highlighting that the achieved battery performance stands out as highly competitive compared to other LSBs documented in the literature with different cathodes (Table S3, Supporting Information).

To meet the high‐energy density requirements of practical applications, high‐sulfur‐loading Li−S cells were further examined under lean electrolyte conditions. The Zn‐Mo_2_C/S‐based Li−S batteries exhibit areal capacities of 5.5 and 7.0 mAh cm^−2^ with sulfur loadings of 6.0 and 8.0 mg cm^−2^, respectively, significantly surpassing the 3.0 mAh cm^−2^ of commercial Li‐ion batteries. Notably, the GCD voltage profiles of these high‐sulfur‐loading cells exhibit two clear plateaus at 0.2 C (Figure 4g). Taking into account key parameters such as sulfur loading, E/S ratio, and areal capacity, this electrochemical performance provides substantial advantages over recently reported state‐of‐the‐art cathode materials (Figure 4h).^[^ ^33^, ^34^, ^35^, ^36^, ^37^, ^38^, ^39^, ^40^ ^]^

Taking advantage of its superior area capacity at high sulfur loading and the straightforward gram‐level production of Zn‐Mo_2_C, flexible pouch cells were assembled with 5.9 mg cm^−2^ sulfur loading, an E/S ratio of 4.2 µL mg^−1^, and a Li anode in 100% excess, to further validate its suitability for commercial use. The Zn‐Mo_2_C‐based Li–S pouch cells exhibit an impressive reversible capacity of 931 mAh g^−1^ and sustain 72.7% capacity retention after 95 cycles (Figure 4j). Notably, the GCD curves (Figure 4i) show consistent performance without significant alteration over hundreds of cycles, affirming their practical effectiveness. The results unequivocally validate the enhanced sulfur trapping and electrocatalytic properties of Zn‐Mo_2_C, which substantially improve sulfur utilization and reaction kinetics in Li−S batteries. Consequently, the above merits endow Zn‐Mo_2_C/S electrode with high specific capacity and exceptional cycling stability.

**Figure 5:**
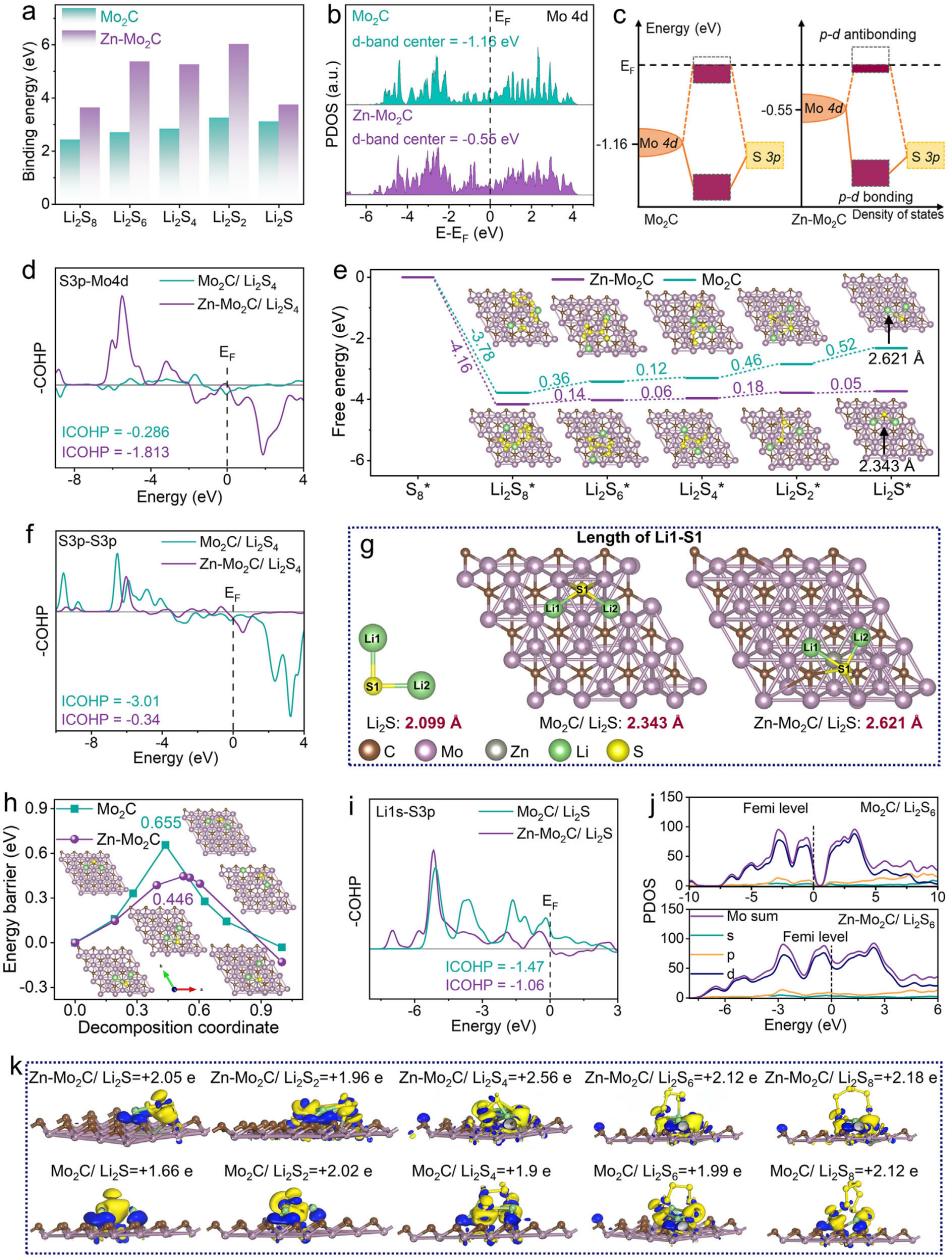
a) Adsorption energy of different polysulfide species (Li_2_S_8_, Li_2_S_6_, Li_2_S_4_, Li_2_S_2_, Li_2_S) on the surface of Mo_2_C and Zn‐Mo_2_C. b) PDOS of Mo_2_C and Zn‐Mo_2_C. c) Schematic representation of the adsorption mechanism. d) ICOHPs of Li_2_S_4_ absorbed with Mo_2_C and Zn‐Mo_2_C, Mo─S bond. e) The Gibbs free energy changes of sulfur reduction processes on Zn‐Mo_2_C and Mo_2_C, show a much lower reaction free energy from Li_2_S_4_ to Li_2_S on Zn‐Mo_2_C than that on Mo_2_C. f) ICOHPs of Li_2_S_4_ adsorbed on the surface of catalysts, S─S bond. g) Geometric models of Li_2_S adsorbed on catalysts. h) Energy profiles for the Li_2_S dissociation process, the inset shows the reaction pathways of Li_2_S decomposition on Zn‐Mo_2_C and Mo_2_C catalysts. i) ICOHPs of Li_2_S adsorbed on the surface of catalysts, Li─S bond. j) PDOS of Li_2_S_6_ adsorbed on Mo_2_C and Zn‐Mo_2_C. k) Charge density difference of LiPSs adsorbed on Zn‐Mo_2_C or Mo_2_C, Blue and yellow represent charge accumulation and loss, respectively.

To explore how Zn doping enhances the adsorption and electrocatalytic activity of Mo_2_C at the atomic level, DFT calculations were conducted. As shown in **Figure** **5**a, the binding energies of Zn‐Mo_2_C to Li_2_S_8_, Li_2_S_6_, Li_2_S_4_, Li_2_S_2_, and Li_2_S are 3.63, 5.37, 5.26, 6.02, and 3.74 eV, respectively, which are higher than those of Mo_2_C (2.42, 2.70, 2.83, 3.25, and 3.11 eV, respectively). To elucidate the enhanced binding energy mechanism, we calculated the density of states (DOS) for Mo_2_C and Zn‐Mo_2_C and identified their d‐band center positions. As shown in Figure 5b, bonding orbitals are located below the Fermi level, and anti‐bonding orbitals are above it. In a crystal field, d orbitals split into bonding and anti‐bonding states. The closer the d‐band center is to the Fermi level (E_F_ = 0), the more d‐electrons favor bonding over anti‐bonding orbitals. As shown in Figure 5b,c, the d‐band center of pristine Mo_2_C is −1.16 eV, which shifts to −0.55 eV upon Zn doping. The doping of Zn atoms elevates the d‐band center position on Mo atoms in Mo_2_C, boosting LiPSs adsorption according to d‐band center theory. Crystal orbital Hamilton population (COHP) analysis was conducted to investigate the bonding between Mo atoms in Zn‐Mo_2_C and S atoms in Li_2_S_4_ (Figure 5d). Spin polarization is mainly reflected at the Fermi level, with the positive part of the curve indicating bonding states and the negative part indicating antibonding states. Both bonding and antibonding state energies increase with Zn doping. The bonding strength is assessed by integrating the COHP (ICOHP) below the Fermi level (E_F_). A smaller ICOHP value indicates a reduction in antibonding orbitals and an increase in bonding orbitals below E_F_, thereby strengthening the bonding between catalysts and LiPSs. The ICOHP values of Mo‐S for Mo_2_C and Zn‐Mo_2_C are −0.286 and −1.813, respectively, suggesting a strengthened Mo‐S bond in Zn‐Mo_2_C/Li_2_S_4_ in contrast to Mo_2_C/Li_2_S_4_.

Charge density difference maps (Figure 5k) offer a deeper understanding of charge distributions, where blue areas represent an increase in charge and purple areas indicate charge depletion. The charge density difference patterns of Li_2_S_6_ adsorbed on the surfaces of Zn‐Mo_2_C and Mo_2_C were illustrated in Figure 5b,c. It is worth noting that the electron cloud density between Li_2_S_6_ and Zn‐Mo_2_C is significantly higher than that between Li_2_S_6_ and Mo_2_C, indicating a stronger interaction with Zn‐Mo_2_C. This observation is consistent with the binding energy values, where Li_2_S_6_ has a higher binding energy with Zn‐Mo_2_C (5.37 eV) than with Mo_2_C (2.7 eV). Similar results are seen for Li_2_S_8_, Li_2_S_4_, Li_2_S_2_, and Li_2_S on Zn‐Mo_2_C, suggesting a stronger interaction between LiPSs and Zn‐Mo_2_C over Mo_2_C.

Aside from the strong binding ability to confine polysulfides, the catalytic effect of Zn‐Mo_2_C was further investigated. Figure 5e shows the relative Gibbs free energy landscape for the discharging process from S_8_ to Li_2_S on the surface of Zn‐Mo_2_C and Mo_2_C. The optimized geometric configurations adsorbed with different LiPSs are shown in the inset of Figure 5e. The initial reduction step of S_8_ to the Li_2_S_8_ shows a spontaneous exothermic reaction on both substrates, whereas the subsequent conversions (Li_2_S_8_ → Li_2_S_6_ → Li_2_S_4_ → Li_2_S_2_ → Li_2_S) are endothermic. The largest Gibbs free energy increase occurs during the conversion from Li_2_S_4_ to Li_2_S segment, suggesting this step as the rate‐limiting for the total discharge process.^[^ ^41^, ^42^ ^]^ Recent studies have recently focused on reducing the energy barrier associated with the Li_2_S_4_→Li_2_S conversion, aiming to accelerate the sluggish kinetics of this critical step.^[^ ^4^, ^43^, ^44^, ^45^ ^]^ Notably, the energy barriers for Li_2_S_4_→Li_2_S_2_ and Li_2_S_2_→Li_2_S on Zn‐Mo_2_C are significantly reduced to 0.18 and 0.05 eV, respectively, far lower than those on pristine Mo_2_C (0.46 and 0.52 eV). This reduction also outperforms several state‐of‐the‐art catalysts, including Zn‐implanted MXene (0.53 and 0.71 eV),^[^ ^6^ ^]^ Co_0.9_Zn_0.1_Te_2_ (0.51 and 1.6 eV),^[^ ^20^ ^]^ MoC (1.79 and 1.76 eV),^[^ ^46^ ^]^ and B‐doped Ni_2_P (1.07 and 0.74 eV).^[^ ^47^ ^]^ In addition, COHP analyses were performed on the S─S bonds across different catalysts. As shown in Figure 5f, the antibonding states of the S─S bond in Li_2_S_4_ on Zn‐Mo_2_C shift downward, filling with more states, while the bonding states shift upward, leading to a less negative COHP of Zn‐Mo_2_C compared to Mo_2_C. This shift, characterized by the filling of antibonding states and the upward movement of bonding states, indicates a weakening (or activation) of the S─S bond on Zn‐Mo_2_C. Consequently, the conversion of Li_2_S_4_ into Li_2_S_2_ is promoted by lowering the chemical bond energy, thereby reducing the activation energy of the reaction.^[^ ^47^, ^48^, ^49^ ^]^ On the grounds of above calculations, the incorporation of Zn can significantly lower the reduction barrier of LiPSs, facilitating Li_2_S precipitation. Besides the Li_2_S_4_ to Li_2_S conversion, the decomposition of Li_2_S is another rate‐limiting step in the sulfur conversion process, both significantly affecting the utilization efficiency of active materials and leading to the formation of dead sulfur. Figure S22 (Supporting Information) and the inset of Figure 5e illustrate the optimized structures of the intermediates on the substrates. As shown in Figure 5g, the pristine Li─S bond length of Li_2_S is 2.099 Å, the Li─S bond lengths of Li_2_S after adsorption on Zn‐Mo_2_C and Mo_2_C increase to 2.343 and 2.621 Å, respectively. The extension of the Li─S bond length in Li_2_S weakens the interatomic bonding, promoting the oxidation of Li_2_S into LiPSs by reducing the chemical bond energy, which in turn decreases the activation energy and accelerates the reduction kinetics of the reaction,^[^ ^50^, ^51^, ^52^ ^]^ aligns well with the experimental results. To further reveal the essential effect of Zn doping on the oxidation kinetics of Li_2_S at the atomic level, the Li_2_S decomposition energy barrier was calculated utilizing the climbing‐image nudged elastic band method (CI‐NEB). The barrier for the Li_2_S oxidation reaction can be derived from the dissociation energy of Li_2_S into LiS and a Li atom on the catalyst, involving multiple diffusion pathways.^[^ ^53^, ^54^ ^]^ As shown in Figure 5h, the calculated energy barriers of Li_2_S dissociation on Mo_2_C and Zn‐Mo_2_C catalysts are 0.655 and 0.446 eV, respectively, indicating that Zn doping exclusively accounts for decreasing the Li_2_S oxidation energy barrier for the charging process. COHP analysis was further employed to investigate the bonding and antibonding characteristics of Li─S bonds in Li_2_S adsorbed on Mo_2_C and Zn‐Mo_2_C catalysts (Figure 5i). For the Zn‐Mo_2_C/Li_2_S model, the ‐ICOHP value of the Li‐S bond is calculated to be −1.06, higher than that for Mo_2_C (−1.47 eV). This comparison suggests that Zn‐Mo_2_C more effectively weakens the Li─S bonds, a conclusion supported by the significant elongation of the Li─S bond within the Zn‐Mo_2_C/Li_2_S system (Figure 5g). The decreased energy barrier for LiPSs redox conversion is linked to two critical factors: 1) The strong Zn─S/Mo─S bonds promote effective d‐p hybridization, weakening nearby Li─S bonds; 2) This weakening makes Li─S bonds more prone to dissociation, thereby reducing the energy barriers.

An electrically conductive phase in cathodes could surely improve the discharge/charge kinetics of Li‐S batteries. The total density of states (DOS) curves reveal that Zn‐Mo_2_C and Mo_2_C are the electrical conductors and valence bands crossing the Fermi level (Figure S23, Supporting Information). Notably, the implantation of Zn atom broadened the DOS around the Fermi level, leading to the increase of the energy levels of the valence band, thereby increasing the conductivity of Mo_2_C. Lithium polysulfides are known for their intrinsic insulating properties, as highlighted in a previous study.^[^ ^55^ ^]^ The conductivity of the substrate/LiPSs after adsorption is critical for efficient sulfur conversion. Therefore, we utilized partial density of states (PDOS) calculations to investigate the electronic structures of the Zn‐Mo_2_C/Li_2_S_x_ and Mo_2_C/Li_2_S_x_ systems. Taking the Li_2_S_6_ system as an example, the inherently insulating Li_2_S_6_ molecule exhibits an electron discontinuity near the Fermi level (Figure S24, Supporting Information). Interestingly, the Zn‐Mo_2_C/Li_2_S_6_ system enhances the electron cloud density at the Fermi level more effectively than the Mo_2_C/Li_2_S_6_ system (Figure 5j). Similar observations can be observed in the Zn‐Mo_2_C/Li_2_S, Zn‐Mo_2_C/Li_2_S_2_, Zn‐Mo_2_C/Li_2_S_4_, and Zn‐Mo_2_C/Li_2_S_8_ systems (Figure S25, Supporting Information). This indicates that the doping of Zn can reduce electrochemical impedance, promote electron transfer between catalysts and LiPSs, and accelerate the reversible sulfur conversion in LSBs.^[^ ^56^ ^]^ The metallic conduction feature significantly accelerates charge transfer of electrocatalyst‐assisted LiPSs redox.^[^ ^57^, ^58^, ^59^, ^60^ ^]^

Catalytic sulfur conversion in lithium–sulfur batteries is a unified process involving three fundamental steps: 1) adsorption of polysulfides onto the catalyst, 2) activation of polysulfides, and 3) desorption of reaction products.^[^ ^48^ ^]^

(3) $$Li_{2}S_{4}+*\;=Li_{2}S_{4}*$$

(4) $$Li_{2}S_{4}*+2Li^{+}+2e^{-}+*\;=2Li_{2}S_{2}*$$

(5) $$2Li_{2}S_{2}*=2Li_{2}S_{2}+2*$$

Mo_2_C captures polysulfides mainly through Mo–S bond formation. When Mo in Mo_2_C interacts with LiPSs, forming Mo─S bonds, the structural configuration of LiPSs transforms, typically resulting in weakened S─S bonds.^[^ ^48^, ^51^ ^]^ Previous studies have established an inverse correlation between the strength of the M─S bond and the S─S bond.^[^ ^48^, ^51^ ^]^ This implies that enhanced adsorption can promote both process (1) and process (2). However, excessively strong adsorption hinders the desorption of reaction products. Zhang et al. demonstrated a volcano‐type relationship between polysulfide adsorption capacity and catalytic activity, wherein excessive adsorption strength impairs the desorption in stage (3), thereby slowing sulfur conversion kinetics.^[^ ^48^ ^]^ This indicates that each catalyst likely possesses an optimal adsorption threshold, beyond which catalytic performance deteriorates. However, the intrinsic adsorption capability of Mo_2_C remains relatively weak,^[^ ^10^, ^62^ ^]^ falling below the threshold required for optimal adsorption. To overcome this limitation, we incorporated Zn doping to raise the d‐band center, shifting specific antibonding orbitals in Mo─S bonds above the Fermi level. This modification significantly enhances Mo_2_C's ability to adsorb LiPSs (confirmed by polysulfide adsorption experiment in Figure 2a and binding energy calculation in **Figure** **5**a) and improves its activation efficiency (supported by S─S bond analysis in ‐ICOHP, Figure 5f). Consequently, Zn‐Mo_2_C further improves the overall catalytic performance.

## 3 Conclusion

In summary, we have developed a hierarchical and conductive Zn‐doped molybdenum carbide microflowers (denoted as Zn‐Mo_2_C) to serve as a sulfur host in LSBs. Zn doping elevates the d‐band center of Mo atoms in Mo_2_C, pushing certain antibonding orbitals above the Fermi level. This change allows for i) enhancing the Mo‐S interaction, effectively anchoring of soluble LiPSs and Li_2_S; ii) The weakening/extending of S─S bonds in the sulfur chain, promoting LiPSs reduction, and the incorporation of Zn significantly reduces the Gibbs free energy barrier for the rate‐limiting step of the Li_2_S_2_ → Li_2_S conversion, from 0.52 eV for Mo_2_C to just 0.05 eV for Zn‐doped Mo_2_C; iii) The stretching of Li–S bonds of Li_2_S, lowering the oxidation energy barrier toward the dissolution of Li_2_S. In addition, this adjustment accelerates charge transfer within the Zn‐Mo_2_C/LiPSs system, thereby significantly enhancing the sulfur conversion kinetics, particularly the rate‐limiting conversion steps. Accordingly, the Zn‐Mo_2_C/S cathodes deliver high rate capability (849 mAh g^−1^ at 2 C) and good cycle stability at 5 C for 1000 cycles (0.021% decay per cycle). The design principles not only enlighten the material design and doping engineering in Li‐S batteries but also provide crucial guidance for related energy storage and conversion materials that simultaneously require electrical, ionic conductivities, and electrocatalytic activity.

## Conflict of Interest

The authors declare no conflict of interest.

## Supporting information



Supporting Information

## Data Availability

The data that support the findings of this study are available from the corresponding author upon reasonable request.
