# Supplementary material for: Zinc‐Doping‐Induced Electronic States Modulation of Molybdenum Carbide: Expediting Rate‐Determining Steps of Sulfur Conversion in Lithium‐Sulfur Batteries
Source: Adv Sci (Weinh). 2025 Mar 31;12(22):2417126. doi: 10.1002/advs.202417126 (PMC12165060; doi:10.1002/advs.202417126)
Supplement: Supplementary file 1 — Supporting Information [file ADVS-12-2417126-s001.docx]

Supporting Information

Zinc-Doping-Induced Electronic States Modulation of Molybdenum Carbide: Expediting Rate-Determining Steps of Sulfur Conversion in Lithium-Sulfur Batteries

Bin Qin,* Yanmei Li, Qun Wang,* Si Zhang, Jinglin Zhang, Bin Wang, Peijia Wang, Yuhan Chen, Weiqi Yao, Fang Wang*

Dr. B. Qin, Y. Li, S. Zhang, J. Zhang, Prof. F. Wang

Key Laboratory of Magnetic Molecules and Magnetic Information Materials of Ministry of Education & School of Chemistry and Materials Science, Shanxi Normal University, Taiyuan 030001, China

Shanxi Key Laboratory of Advanced Magnetic Materials and Devices & Research Institute of Materials Science of Shanxi Normal University, Taiyuan 030001, China

E-mail: bqin2020@163.com, wangfanghc@sxnu.edu.cn

Dr. Q. Wang

Basic Department

Shanxi Agricultural University

Jinzhong, 030801, China

E-mail: wangqun0565@163.com

Dr. B. Wang, Dr. P. Wang

School of Materials Science and Engineering

Harbin Institute of Technology

Harbin, 150001, China

Dr. Y. Chen

Department of Mechanical Engineering

City University of Hong Kong

Hong Kong SAR, China

Dr. W. Yao

Materials Science and Engineering Program & Texas Materials Institute

The University of Texas at Austin

Austin, Texas 78712, United States

**Supporting experimental section**

**Material synthesis and sulfur cathode fabrication**

*Synthesis of* *ZnMoO_4_ microflowers*. All chemicals employed in this study were of analytical grade and utilized without any further purification. Commercial molybdenum carbide (99.95% purity) was purchased from Aladdin. To initiate the experimental procedure, 2.5 mmol Zn(NO_3_)_2_·6H_2_O, 2.5 mmol Na_2_MoO_4_·2H_2_O, 5 mmol CO(NH_2_)_2_, and 5 mmol NH_4_F were dissolved in a mixed solution of 62.5 mL water and 12.5 mL ethanol, followed by 3 h of magnetic stirring. The resultant solution was transferred to a stainless steel autoclave and heated in an oven at 160 °C for 12 h. Upon natural cooling to room temperature, the product was filtered, rinsed with deionized water, and dried to obtain white ZnMoO_4_ powder.

*Synthesis of Zn-Mo_2_C*. Dicyandiamide (C_2_H_4_N_4_) and the as-synthesized ZnMoO_4_ powder were placed in the upstream and downstream sections of a tube furnace, respectively. The furnace was heated to 750~850 °C at a rate of 5 °C min^−1^ under an argon atmosphere and maintained at this temperature for 2 h, as demonstrated in the schematic illustration of Figure S1. After natural cooling, Zn-Mo_2_C powder was obtained. Notably, unless otherwise specified, Zn-Mo_2_C refers to the material synthesized at a thermal treatment temperature of 800 °C.

*Synthesis of sulfur cathodes*. A mixture of 70 wt.% sublimed sulfur and 30 wt.% Zn-Mo_2_C was subjected to a thermal process at 155 °C for 12 h within an ampoule. Following this process, the mixture was dispersed in NMP, combined with Super P and PVDF in a weight ratio of 8: 1: 1 to produce a uniform slurry. This slurry was coated onto aluminum foil and dried under vacuum at 120 °C for 24 h. The resulting 12 mm diameter disks served as sulfur composite cathodes (labeled as Zn-Mo_2_C/S). For standard electrochemical measurements, the areal loading of the Zn-Mo_2_C/S composite cathode was around 2.0 mg cm^−2^. Higher sulfur loading electrodes (6.0 and 8.0 mg cm^−2^) were also prepared using the LA133 binder to ensure mechanical stability, adhering to the same preparation procedure.

**Materials characterizations**

Morphological characteristics were examined using scanning electron microscopy (SEM) and transmission electron microscopy (TEM) with Merlin Compact, Zeiss Helios, and Tecnai G2 F30 (200 kV) instruments, respectively. Raman spectra were recorded with an ATR8300MP system using a 532 nm laser. X-ray diffraction (XRD) analysis was conducted using a D/max 2550 instrument with Cu Kα radiation (λ = 1.5406 Å) at 30 kV and 10 mA. The composition and valence state of the samples were analyzed by X-ray photoelectron spectroscopy (XPS) using a ThermoFisher ESCALAB 250Xi system. UV–visible spectra within the 250–700 nm range were obtained using a Cary 60 UV–vis spectrophotometer. The nitrogen adsorption–desorption isotherms were collected on an ASAP (2020)M apparatus at 77 K.

**Assembly of lithium-sulfur batteries and electrochemical measurements**

After preparing the sulfur cathodes, we assembled CR2032 coin-type batteries. These batteries included a Celgard 2500 polypropylene (PP) separator, a lithium foil anode, and a commercial electrolyte composed of 1.0 M LiTFSI in a 1:1 volume ratio of DME and DOL, with 2% LiNO_3_ additive. All assembly steps were performed in an argon-filled glove box with H_2_O/O_2_ levels maintained below 0.1 ppm.

Galvanostatic charge-discharge (GCD) measurements were conducted between 1.7 and 2.8 V using a LAND 2100CT battery testing system at various current densities (1 C = 1675 mAh g^−1^) at room temperature. Cyclic voltammetry (CV) tests were executed using a CHI 760E electrochemical workstation. For battery performance evaluation, GCD measurements were systematically carried out at different current densities on a LAND 2100CT system, with 1 C defined as 1675 mAh g^−1^, under ambient conditions.

**Visualized adsorption and ultraviolet-visible (UV-vis) measurements**

For the preparation of a 5 mM Li_2_S_6_ solution, lithium sulfide, and sublimed sulfur were dissolved in a 1:5 molar ratio in a 1:1 volumetric mixture of DOL and DME solvents. Prior to adsorption tests, commercial Mo_2_C, Super P, and Zn-Mo_2_C powders were dried under vacuum at 70 °C for 12 h. Equal surface area (set as 0.6 m^2^, equal to that calculated from the product of the surface area and sample weight) of each powder were then added to the Li_2_S_6_ solution. After a gentle shake for 10 s, the mixture was left undisturbed for 6 h. During this period, digital photographs were taken to visually record the adsorption process. The entire test was performed inside an argon-filled glove box. After the test, the supernatants were diluted with DME/DOL solvent at a 1:100 ratio to enable quantitative concentration analysis via UV–vis spectroscopy. The precipitated products were collected and dried for XPS analysis.

**Measurements for Li_2_S nucleation and** **dissociation**

To investigate the kinetics of liquid-solid conversion, a 0.2 M Li_2_S_8_ catholyte was prepared by stirring sulfur and Li_2_S in tetraglyme solution at 60 °C for 24 h, maintaining a 7:1 molar ratio. Li_2_S Nucleation and dissociation experiments were performed in standard 2032 coin cells. The synthesized Zn-Mo_2_C and commercial Mo_2_C hosts were sectioned into 12 mm diameter wafers and used as the working electrodes. Lithium foil served as the counter electrode, and Celgard 2500 was used as the separator. In standard 2032 coin cells, nucleation of Li_2_S on matrixes was systematically investigated. The procedure involved adding 20 μL of Li_2_S_8_ solution to the cathode and an equal volume (20 μL) of blank Li-S electrolyte to the anode. Assembled cells were initially discharged galvanostatically at 0.112 mA up to 2.06 V, consuming most high-order polysulfides. A potentiostatic discharge was then performed at 2.05 V to collect the Li_2_S precipitation current until it dropped below 10^−5^ A. Time-current curves were recorded to study Li_2_S nucleation capacities using Faraday's law. For Li_2_S oxidation tests, the batteries were first discharged galvanostatically at 0.05 C to 1.7 V to generate Li_2_S. Subsequently, a potentiostatic charging at 2.4 V was performed to dissociate Li_2_S into LiPSs until the current dropped below 10^−5^ A.

**Fabrication and measurement of symmetric cells**

Symmetric cells were assembled using two identical hosts with a Celgard 2500 membrane as the separator. The electrolyte chosen for this setup was 40 µL of 0.2 M Li_2_S_6_. To explore the catalytic properties, cyclic voltammetry (CV) and electrochemical impedance spectroscopy (EIS) measurements were systematically performed. CV tests were conducted with a CHI 760e instrument, cycling the voltage from −0.7 to 0.7 V at a scan rate of 15 mV s^−1^. EIS measurements were carried out on a PARSTAT 2273 electrochemical workstation, covering frequencies from 10 mHz to 100 kHz with an amplitude of 10 mV.

**Self-discharge measurements**

To investigate the self-discharge behavior of the cells, the open circuit voltage (OCV) of coin cells was consistently monitored throughout the self-discharge measurements. Initially, the batteries underwent 52 discharge and charge cycles, reaching full charge at 2.8 V, with each cycle conducted at 0.5 C. Following this, the batteries were rested for 72 h before undergoing an additional 108 cycles of continuous cycling.

**Shuttle current tests**

For shuttle current tests, a LiNO_3_-free electrolyte was used to prevent passivation of the Li anode. Initially, the cells underwent three charge-discharge cycles before being galvanostatically charged to 2.8 V at a rate of 0.2 C. After about 10^4^ s, the potentiostatic current reached a steady state, allowing for accurate recording of the shuttle current.

**Pouch cell assembly and measurements**

To assemble the pouch cell, an areal sulfur loading of approximately 6.1 mg cm^−2^ was achieved for the Zn-Mo_2_C cathode. A 0.1 mm thick Li foil was calendered onto a Cu foil, and both the composite cathode and lithium anode were cut into 5 × 4 cm^2^ pieces. The PP separator and electrolyte were strategically positioned between the Zn-Mo_2_C cathode and Li foil, forming a sandwich configuration. A meticulously controlled E/S ratio of 4.9 μL mg^−1^ was established, with the Li anode maintained at 100% excess. Pouch cell measurements were carried out under the same conditions as those for the CR2032 coin cells.

***In*-*situ* Raman measurements**

For *in*-*situ* Raman spectroscopy characterization, Li-S cells were thoughtfully designed with a quartz window in the negative shell. A 5 mm diameter hole was incorporated to allow precise laser focus on the separator at the anode side. The cells were systematically operated at 0.2 C throughout the experiments, and Raman signals were recorded using a Raman microscope (ATR8300MP).

**Theoretical computations**

We have employed the first-principles to perform density functional theory (DFT) calculations within the generalized gradient approximation (GGA) using the Perdew-Burke-Ernzerhof (PBE) formulation. The projected augmented wave (PAW) potentials were used to account for the ionic cores and incorporate valence electrons. The surface of Mo_2_C (001) had a vacuum thickness of 15 Å in the vertical direction to avoid interaction between the slabs. The kinetic energy cutoff for plane wave expansion was set at 540 eV. The convergence thresholds for energy and atomic forces were set as 10^−5^ eV and 0.02 eV Å^−1^. The Brillouin zone integration was sampled with 3×3×1 Monkhorst-Pack k-point in the Gramma-centered grids for the structure relaxation. The decomposition barriers for Li_2_S dissociation on Mo_2_C were calculated by the climbing image-nudged elastic band (CI-NEB) method. The charge density difference of the Mo_2_C/Li_2_S_n_ (n = 1, 2, 4, 6, 8) model shows the charge accumulation (blue) and depletion (yellow), respectively.

The adsorption energies (E_ads_) were calculated as follows:

*E*_ads_=*E*_substrate+LiPSs_-*E*_substrate_-*E*_LiPSs_ (1)

In the formula, *E*_substrate+LiPSs_ is the total energy of the polysulfide on the surface of the substrate system, *E*_LiPSs_ is the energy of the isolated polysulfide, and *E*_substrate_ is the energy of the clean substrate.

The relative free energy of the discharging reaction from Li_2_S_8_ to Li_2_S on the Mo_2_C and Zn-doped Mo_2_C were calculated by the equations:

ΔG_1_ = G(Li_2_S_6_*) - G(Li_2_S_8_*) + 2G(Li_2_S) - 4G(Li^+^) (2)

ΔG_2_ = G(Li_2_S_4_*) - G(Li_2_S_6_*) + 2G(Li_2_S) - 4G(Li^+^) (3)

ΔG_3_ = G(Li_2_S_2_*) - G(Li_2_S_4_*) + 2G(Li_2_S) - 4G(Li^+^) (4)

ΔG_4_ = G(Li_2_S*) - G(Li_2_S_2_*) + G(Li_2_S) - 2G(Li^+^) (5)

Supporting figures and tables


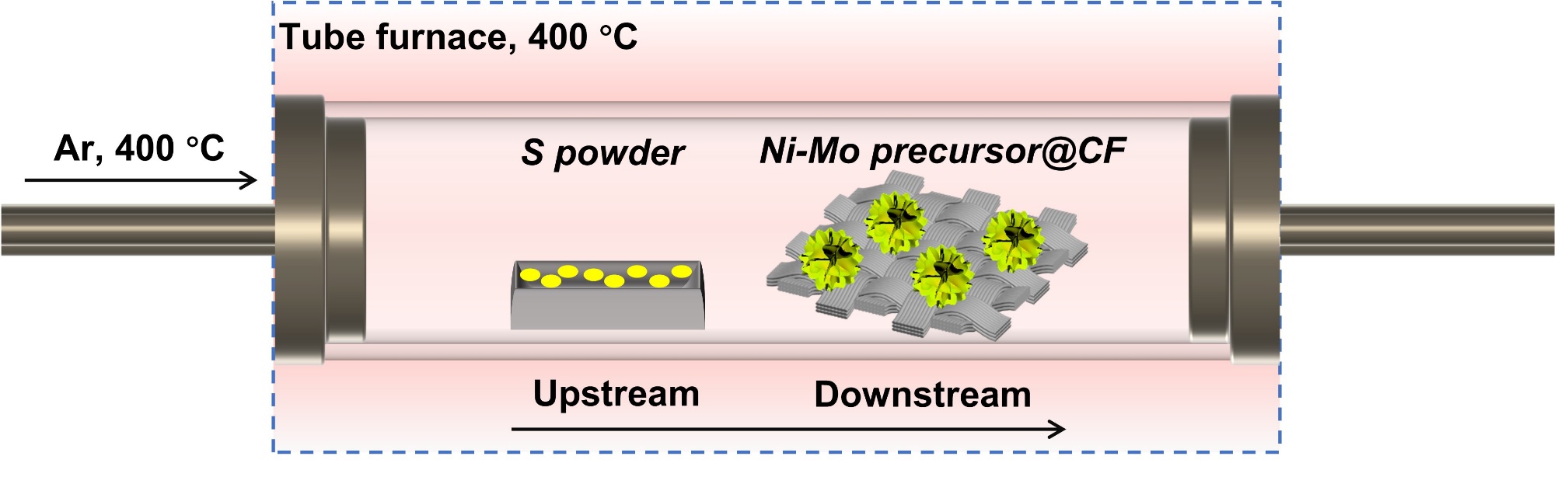


**Figure S1.** Schematic illustration for the synthesis of Zn-Mo_2_C in a tube furnace.

**Figure S2.** XRD pattern of ZnMoO_4_.

**Figure S3.** XRD pattern of Zn-Mo_2_C.


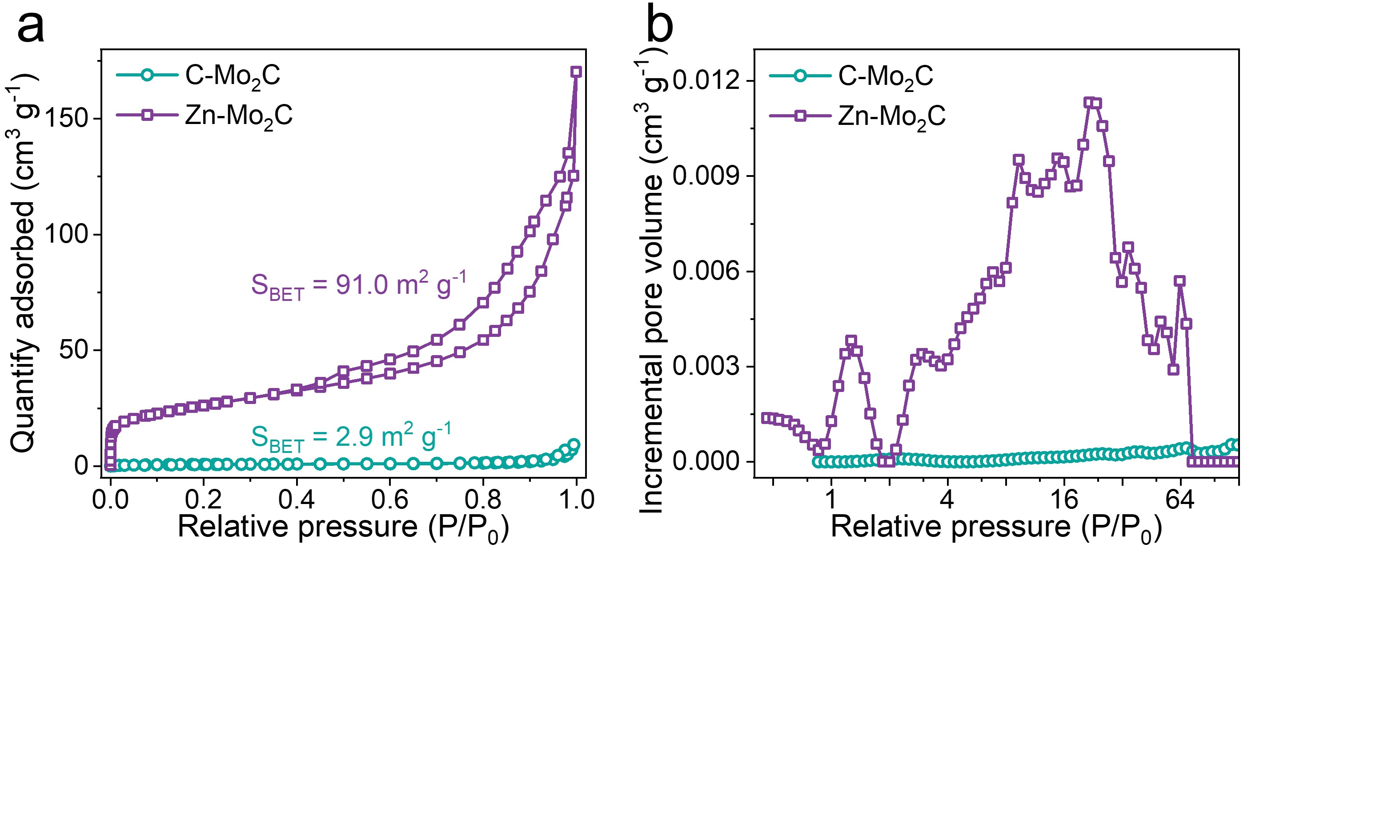


**Figure S4.** (a) Nitrogen adsorption−desorption isotherms and (b) the corresponding pore-size distribution curves (inset) of Zn/Mo_2_C and C-Mo_2_C.

Brunauer−Emmett−Teller surface area (S_BET_) of the samples were analyzed by nitrogen adsorption−desorption measurements. All the samples were degassed at 200 °C under vacuum for 6 h prior to the tests. The pore size distribution was determined through DFT method using the adsorption databased on a slit pore model. Total pore volume (V_t_) was investigated from the adsorbed amount at a relative pressure P/P_0_=0.95. As shown in Figure S4, the S_BET_ and V_t_ of Zn-Mo_2_C are 91.0 m^2^ g^−1^ and 0.394 cm^3^ g^−1^, which is significantly larger than that of C-Mo_2_C (S_BET_ = 2.9 m^2^ g^−1^, V_t_ = 0.014 cm^3^ g^−1^). The large specific surface area of Zn-Mo_2_C can provide more adsorption and catalytic sites for lithium polysulfide. In addition, the larger pore volume of Zn-Mo_2_C can also accommodate more active sulfur, which helps to achieve high sulfur content/loading and high energy density.


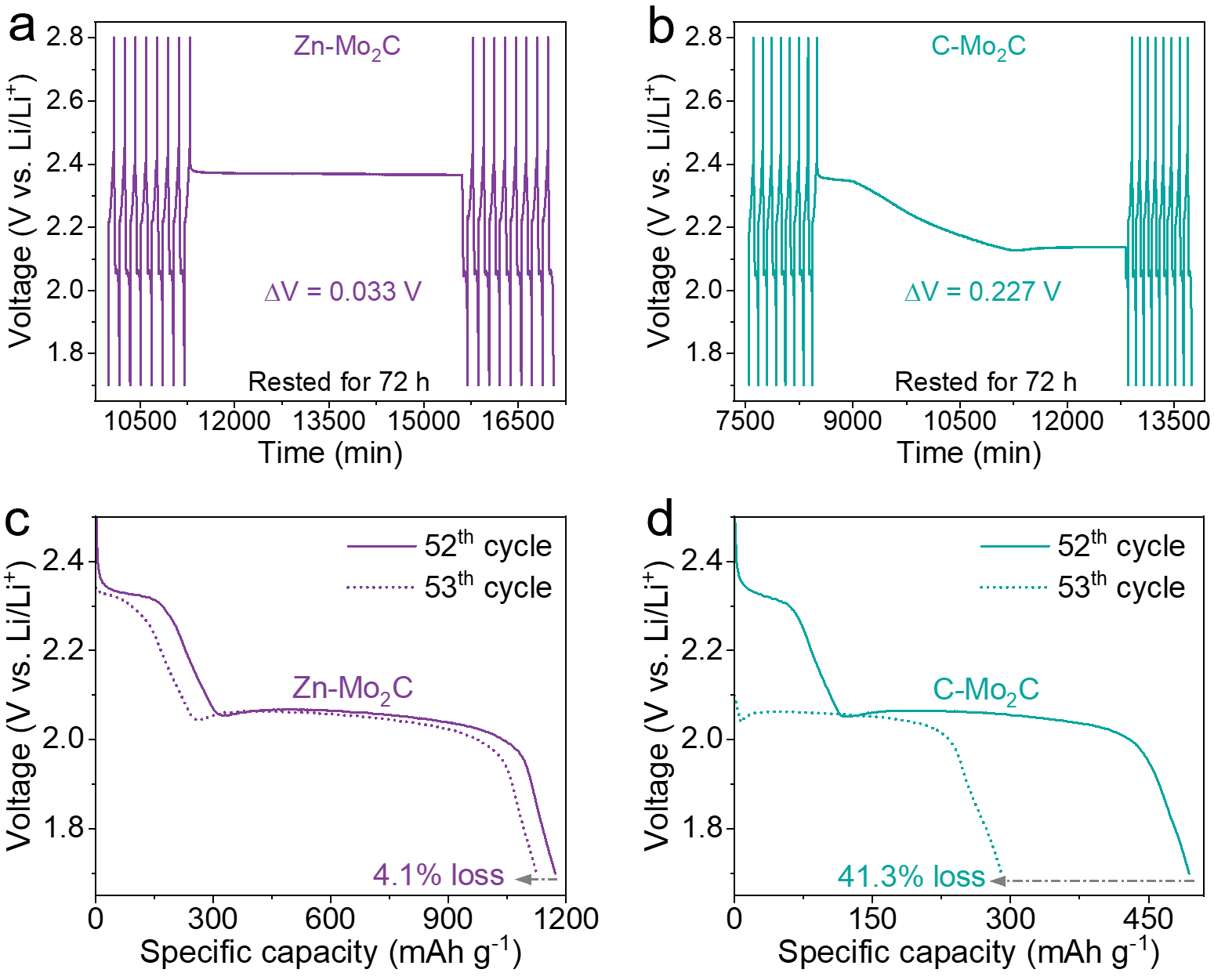


**Figure S5.** Voltage-time curves during the interruption processes of Li-S batteries paired with routine (a) Zn-Mo_2_C and (b) C-Mo_2_C cathodes. Discharge curves and self–discharge behavior of cells with (c) Zn-Mo_2_C and (d) C-Mo_2_C.


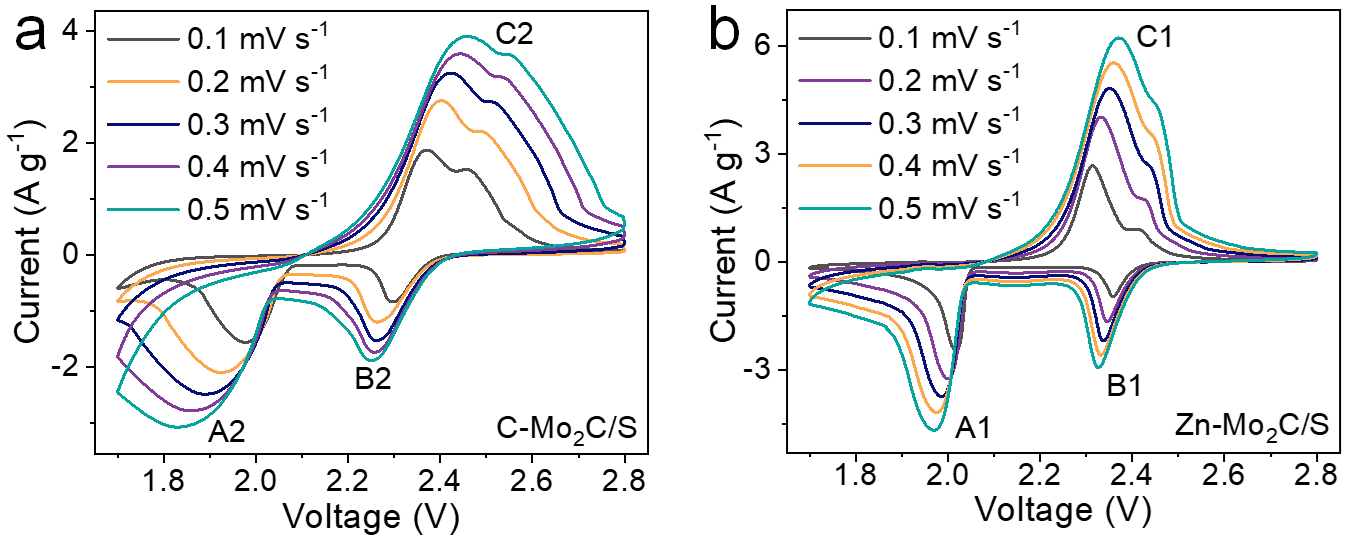


**Figure S6.** CV curves of the (a) C-Mo_2_C/S and (b) Zn-Mo_2_C/S electrodes at different scan rates.


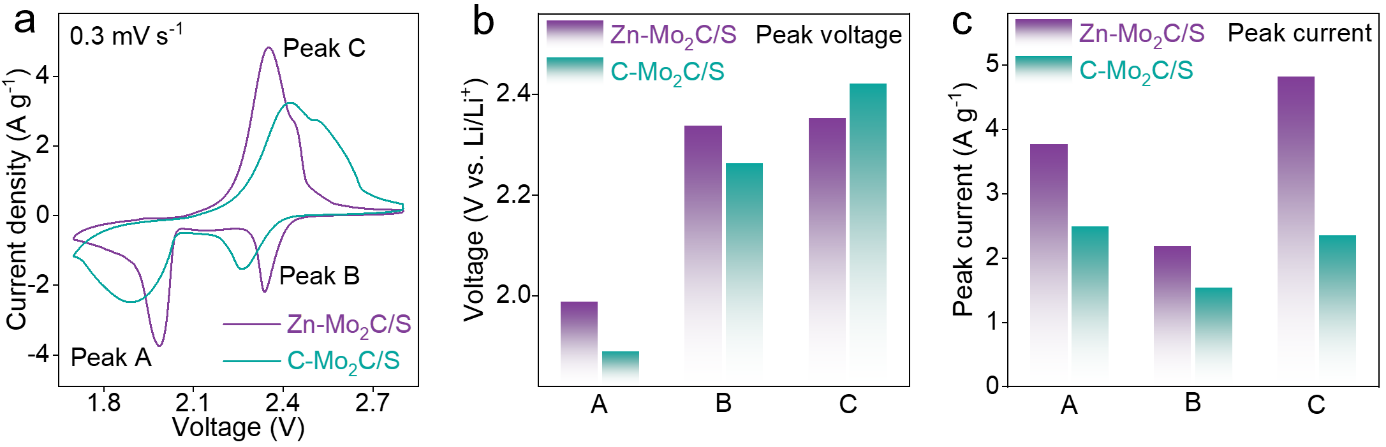


**Figure S7.** (a) CV curves of the Li–S batteries using Zn-Mo_2_C/S and C-Mo_2_C/S as catalysts, 0.3 mV s^−1^. (b) Peak voltages and (c) peak currents obtained from the CV curves with different electrodes.


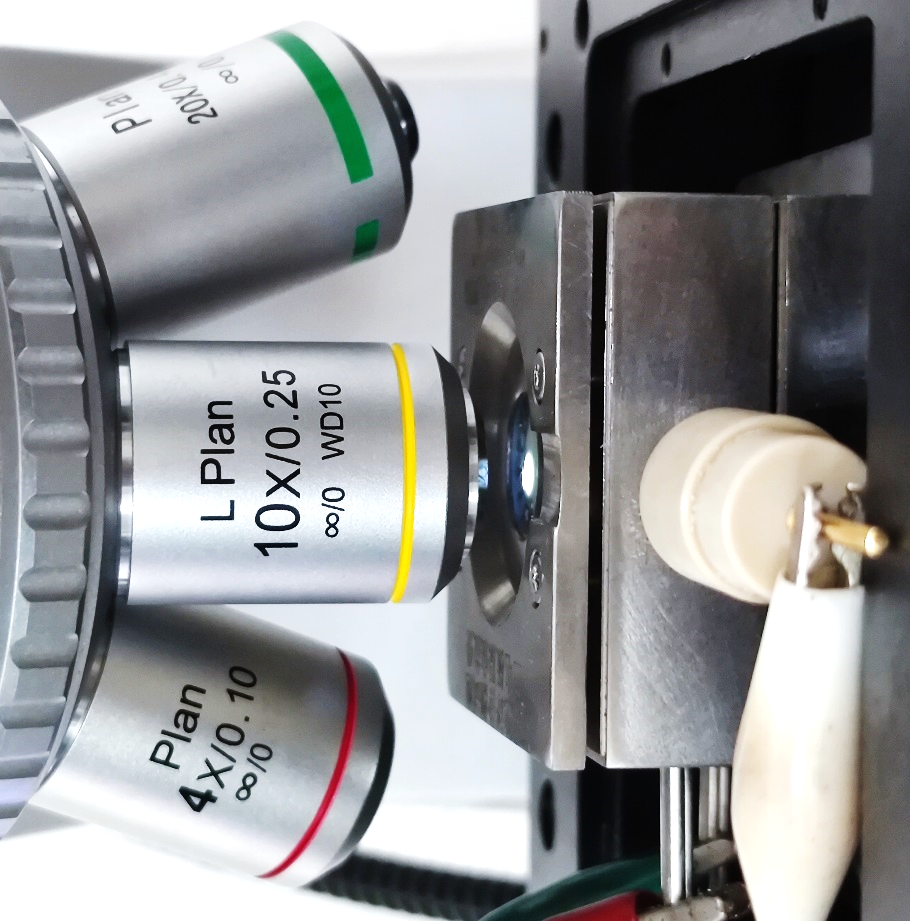


**Figure S8.** Digital photographs of an *in*-*situ* Raman equipment.

**Figure S9.** Shuttle current curves of the cells paired with Zn-Mo_2_C and C-Mo_2_C cathodes.

The shuttle current of Zn-Mo_2_C/S is ≈ 4.4 × 10^−3^ mA cm^−2^, which is significantly lower than that of C-Mo_2_C/S (11.1 × 10^−3^ mA cm^−2^). This proves that the synergistic effect of Zn doping and hierarchical conductive network could significantly confine polysulfides and suppress the shuttle effect of LiPSs during the charging/discharging process.


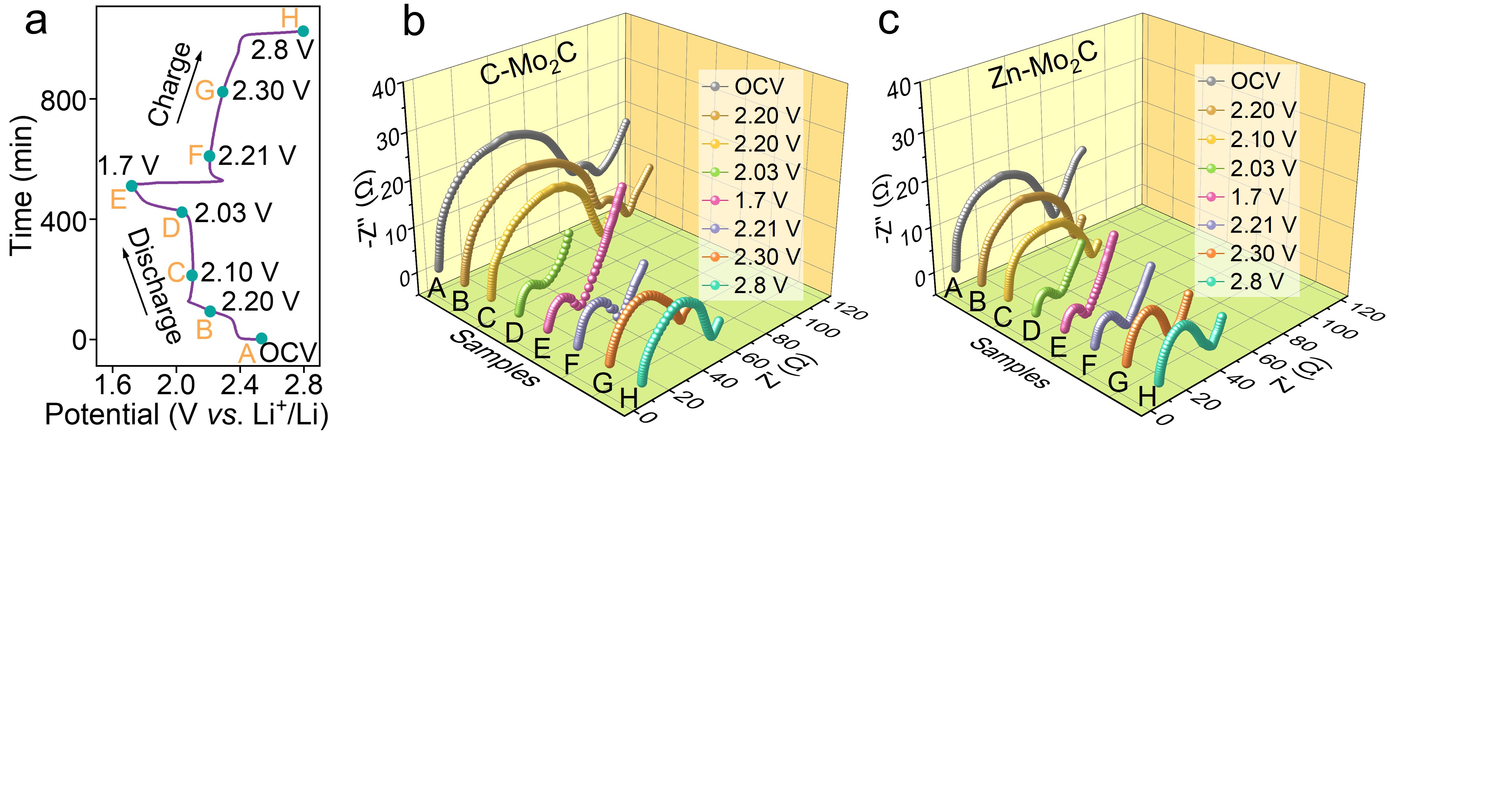


**Figure S10.** (a) Charge/discharge profiles and (b,c) ex-situ EIS spectra for the Zn-Mo_2_C cell at various states of charge and discharge.


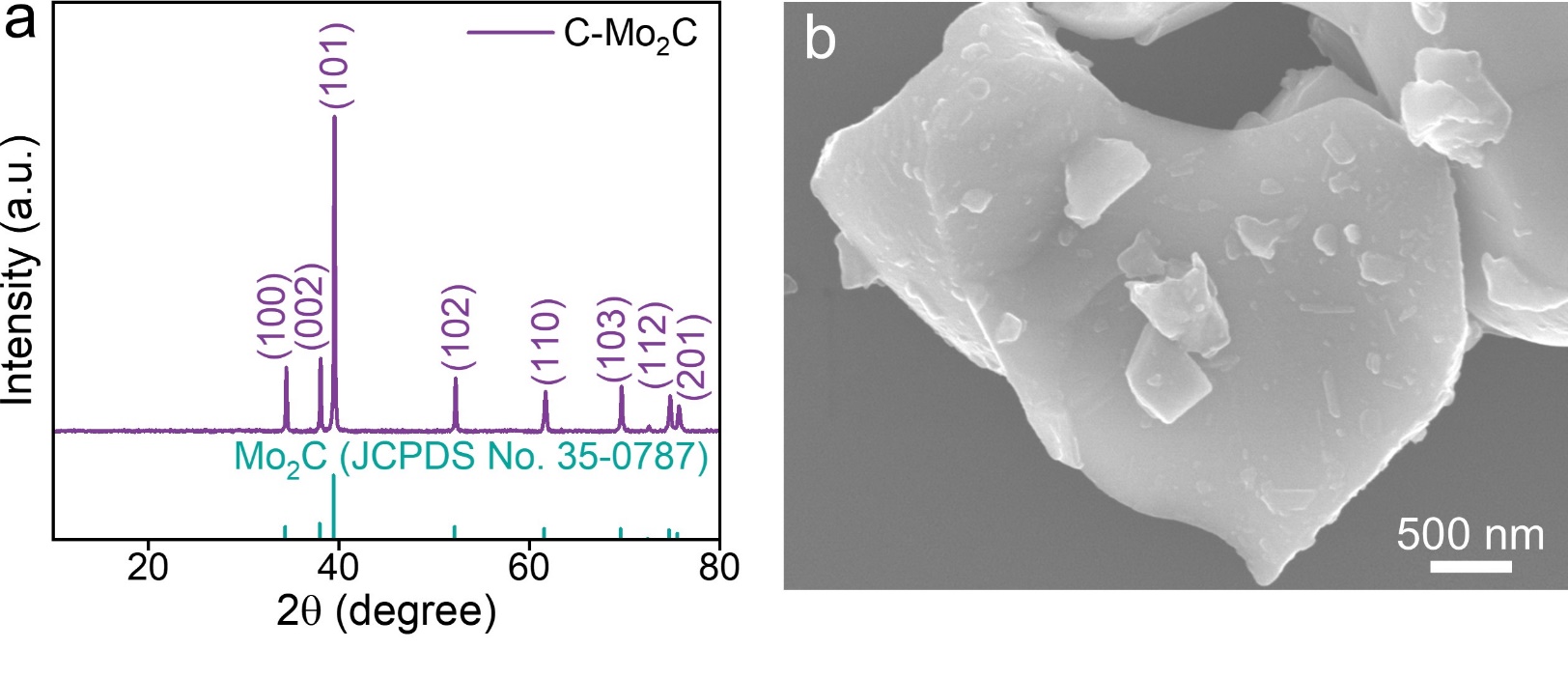


**Figure S11.** XRD result and SEM image of commercial Mo_2_C (C-Mo_2_C).

To demonstrate the advantage of Zn-Mo_2_C with high-density active sites and enhanced catalytic activity for regulating polysulfides conversion, commercial Mo_2_C (C-Mo_2_C) was used as a control electrode. As shown in Figure S11b, C-Mo_2_C shares the same physical phase as Zn-Mo_2_C. Nevertheless, C-Mo_2_C consists predominantly of micrometer-sized particles, which greatly reduces the number of catalytic and adsorptive active sites compared to Zn-Mo_2_C.


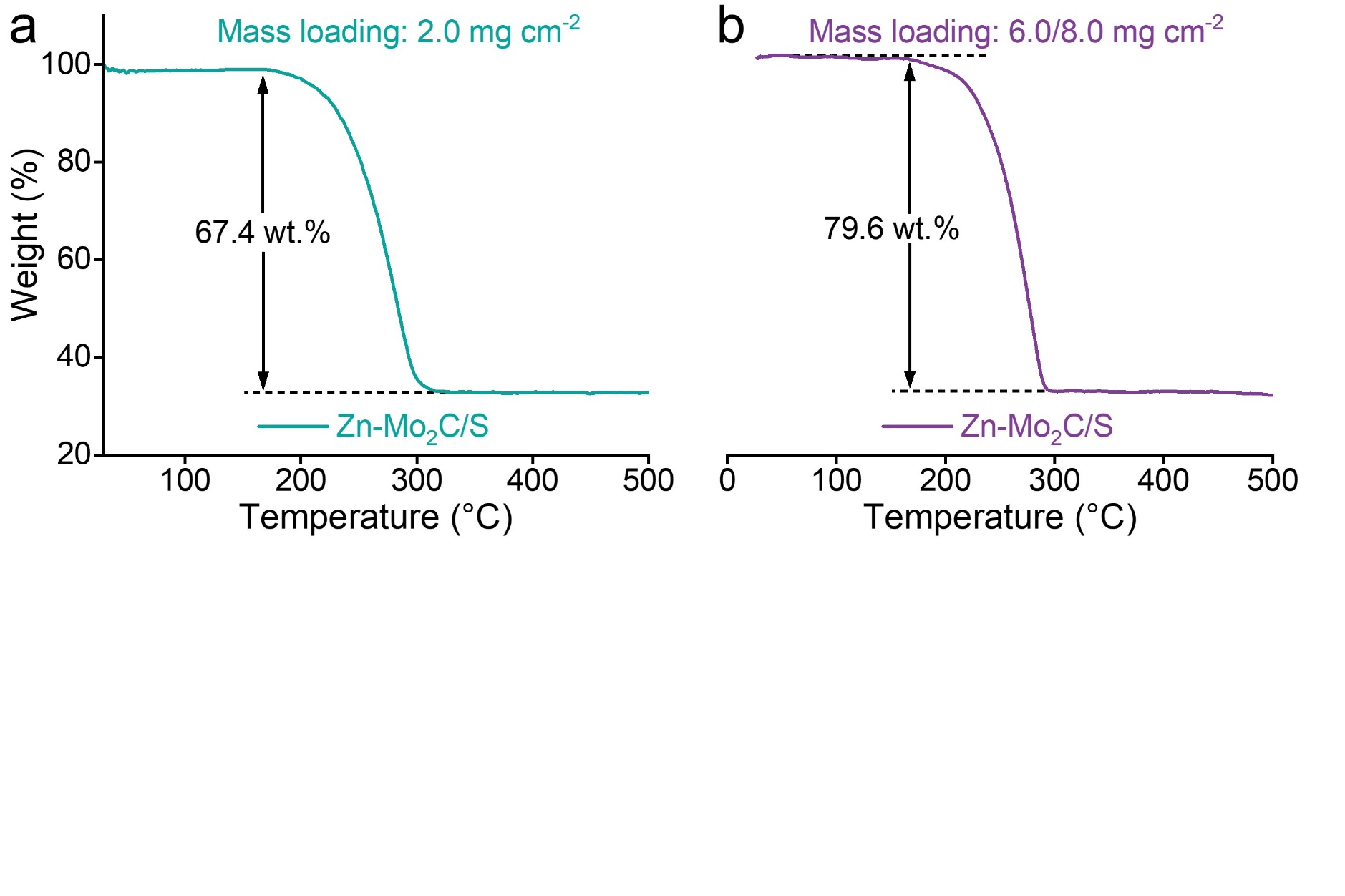


**Figure S12.** TGA curve of Zn-Mo_2_C/S under argon flow.

Figure S12a presents the thermogravimetric (TG) analysis, showing that the conventional Zn-Mo_2_C/S composite cathode (~2.0 mg cm^−2^) contains approximately 67.4 wt.% sulfur. The composite was synthesized using a conventional thermal-diffusion approach and then used as sulfur cathodes in 2032 coin cells. It is worth noting that the S contents in high-sulfur-loading electrodes (6.0 and 8.0 mg cm^−2^) are ~79.6 wt.%, as depicted in Figure S12b.


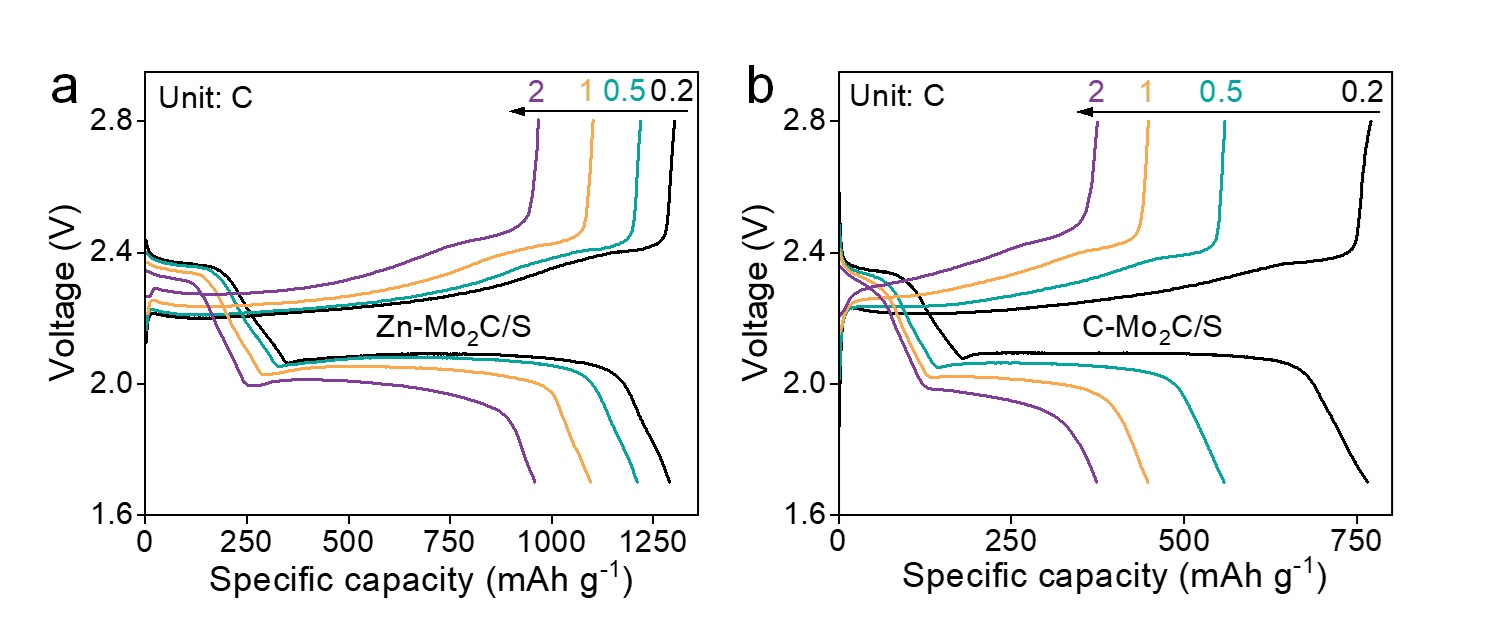


**Figure S13.** Discharge–charge curves of the (a) Zn-Mo_2_C and (b) C-Mo_2_C at various current rates.


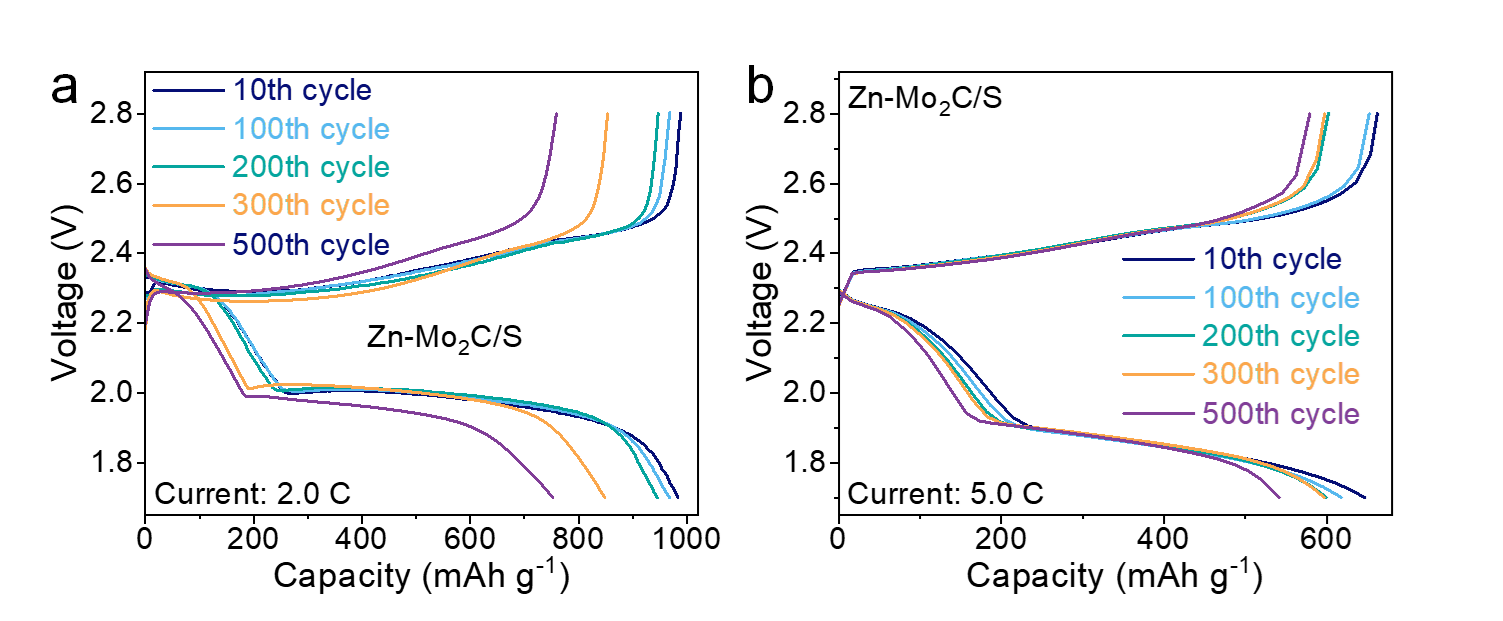


**Figure S14.** Discharge-charge voltage curves of long-term cycles for Zn-Mo_2_C/S electrode at (a) 2.0 C and (b) 5.0 C.


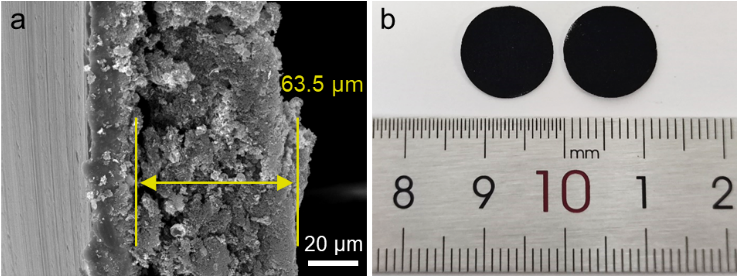


**Figure S15.** (a) Cross-sectional SEM images of Zn-Mo_2_C/S cathode under sulfur loading of 6.0 mg cm^–2^. The corresponding size of the doctor blade is typically 450 μm. (b) The photograph of the cathode. The thicknesses of the Zn-Mo_2_C/S coating layers are around 63.5 µm under sulfur loadings of 6.0 mg cm^−2^.


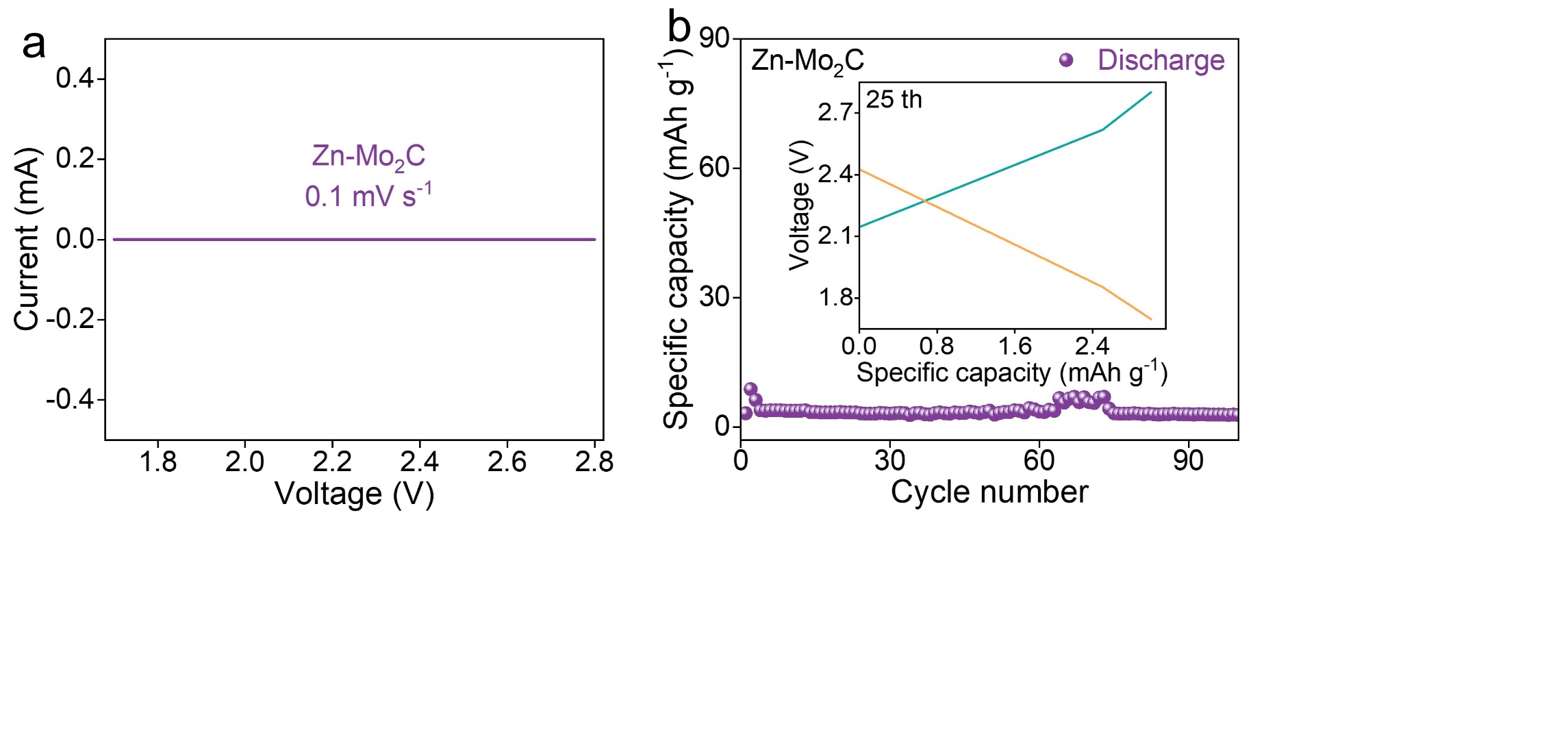


**Figure S16.** (a) CV curve of the pristine Zn-Mo_2_C composite (without sulfur loading) at a scan rate of 0.1 mV s^−1^. (b) Cycling performance of Zn-Mo_2_C at 0.2 C, inset shows the GCD profiles within a voltage window of 1.7-2.8 V (*vs*. Li/Li^+^). Note that the tested discharge capacity of Zn-Mo_2_C is very low and thus exerts little contribution to the total capacity of LSBs.

**Figure S17.** Cycling performance of Zn-Mo_2_C cell in LiNO_3_-free electrolyte.

LiNO_3_, a conventional electrolyte additive, is typically used to suppress dendrite growth caused by side reactions between migrated LiPSs and Li metal.^[1]^ To further investigate the efficacy of the Zn-Mo_2_C host in preventing polysulfide migration, the cycling performance of Zn-Mo_2_C/S batteries was assessed in a LiNO_3_-free electrolyte. As shown in Figure S15, the Zn-Mo_2_C/S cathode delivered an initial capacity of 1210.2 mAh g^−1^ at 0.5 C, and maintained a specific capacity of 993.4 mAh g^−1^ after 150 charge/discharge cycles. Remarkably, the Coulombic efficiency of the Zn-Mo_2_C/S cell was only marginally reduced compared to cells containing 0.2 M LiNO_3_ electrolyte, underscoring the Zn-Mo_2_C matrix’s role in mitigating the shuttle effect in LSBs.


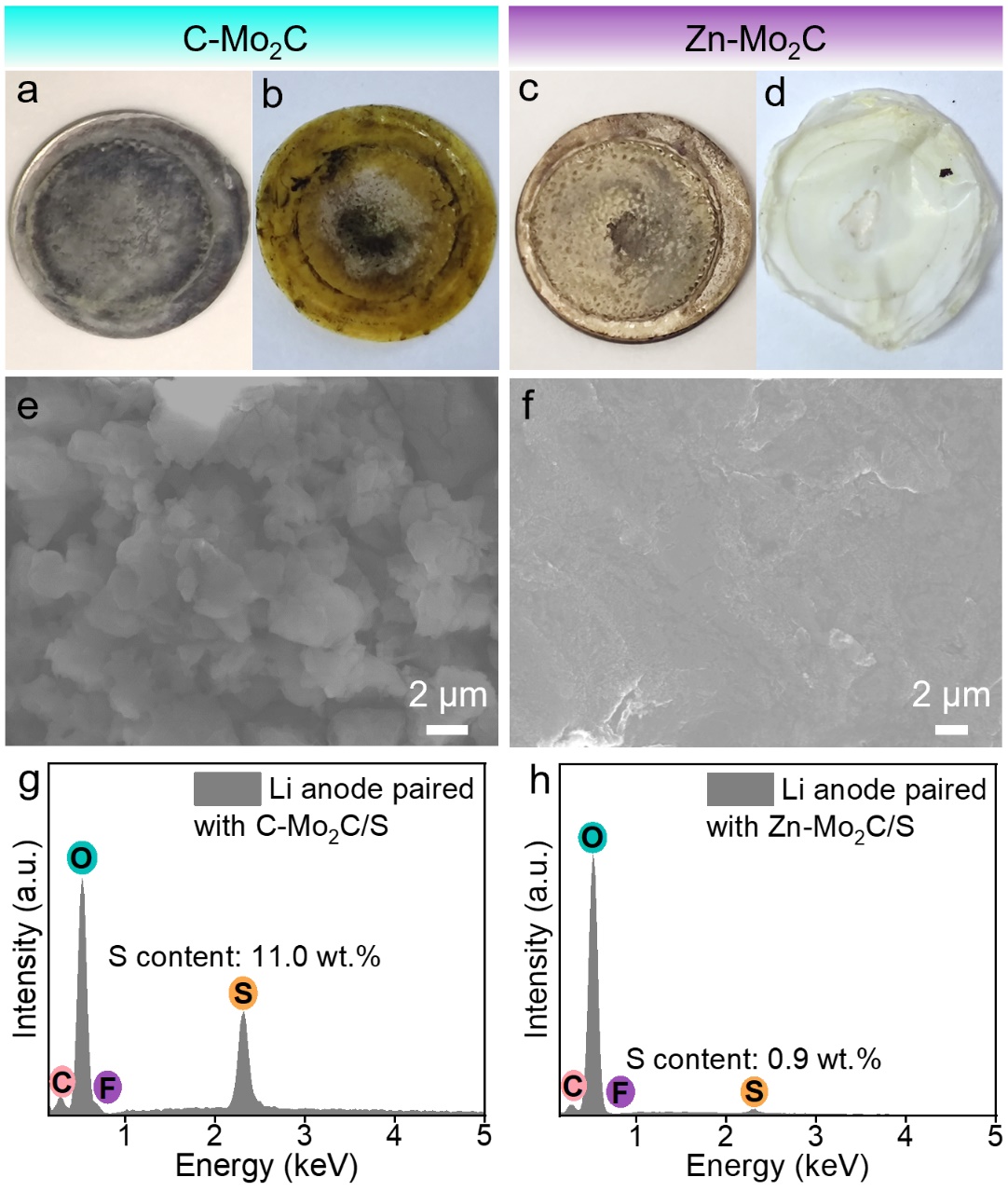


**Figure S18.** Post-mortem analysis of Li-S cells after 150 cycles at 0.5 C. (a,c) Digital images of Li anodes after cycling. (b,d) Optical images of the separators after cycling. (e,f) SEM images of the cycled lithium foils. (g,h) EDS profiles of the cycled Li anodes. Among these hosts, the cell paired with Zn-Mo_2_C delivers a relatively compact surface with the lowest sulfur content, demonstrating the successful confinement of LiPSs.

**Figure S19.** The 150th charge-discharge profiles at 0.5 C.

Evaluating the polarization potential of cycled electrodes offers crucial insights into the battery’s reversibility over extended cycling. As illustrated in Figure S19, after 150 charge–discharge cycles, Zn-Mo_2_C demonstrates a polarization potential of 216.7 mV, considerably lower than the 343.5 mV of C-Mo_2_C, further confirming its superior cycling stability and reversibility.

**Figure S20.** Rate capability of Zn-Mo_2_C samples synthesized at various annealing temperatures.

The annealing temperature affects the Zn doping level in Zn-Mo_2_C. To explore the impact of Zn doping content on electrochemical performance, we systematically studied the Zn content variations in samples prepared under different thermal annealing conditions and its correlation with electrochemical properties. Inductively coupled plasma-optical emission spectroscopy (ICP-OES) analysis reveals that the Zn contents in Zn-Mo_2_C (750), Zn-Mo_2_C (800), and Zn-Mo_2_C (850) are 3.9 wt.%, 2.1 wt.%, and 1.2 wt.%, respectively, indicating a gradual decrease in Zn doping with increasing annealing temperature. Figure S20 compares the rate performance of these three samples, Zn-Mo_2_C (800) achieves the highest reversible capacities at 0.1, 0.2, 0.5, 1, and 2 C among these batteries. Therefore, Zn-Mo_2_C (800) (short labeled as Zn-Mo_2_C in MS) is selected as the primary material for this study.


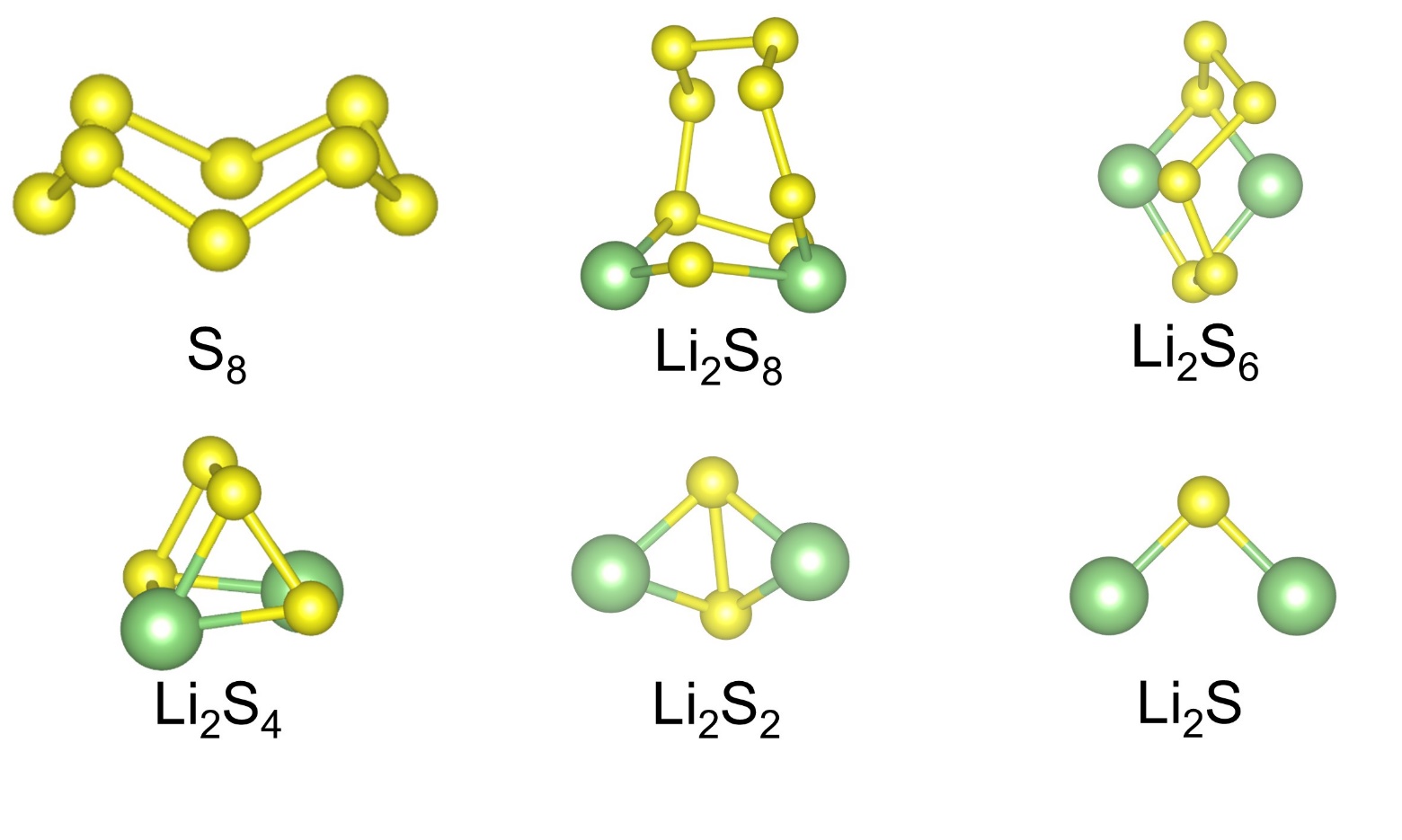


**Figure S21.** Optimized geometries of isolated S_8_, Li_2_S_8_, Li_2_S_6_, Li_2_S_4_, Li_2_S_2_ and Li_2_S.


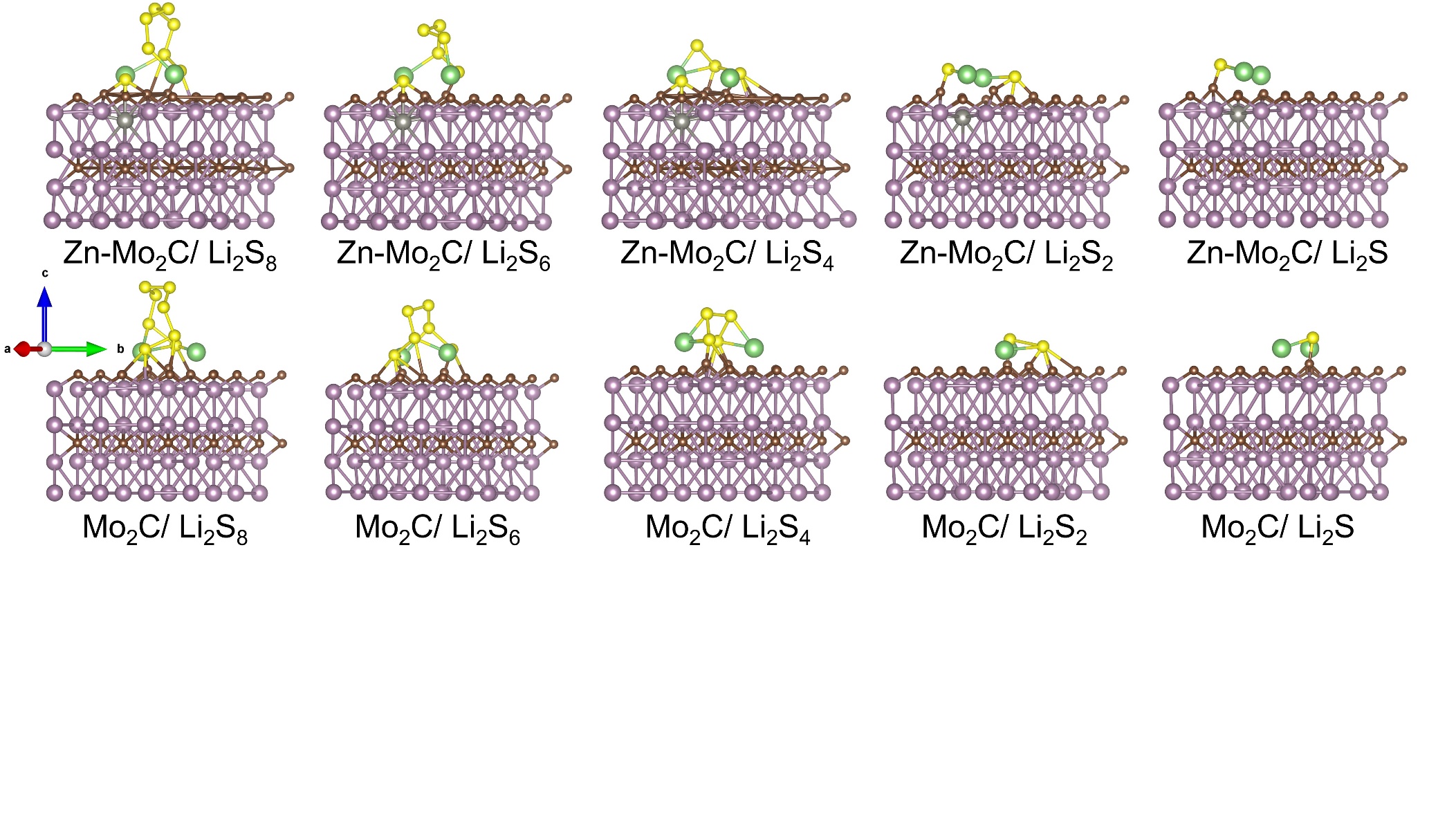


**Figure S22.** Adsorption configurations (side view) of sulfur species on Mo_2_C and Zn-Mo_2_C.

**Figure S23.** Density of states for Mo_2_C and Zn-Mo_2_C.

**Figure S24.** Density of states for Li_2_S_6_.


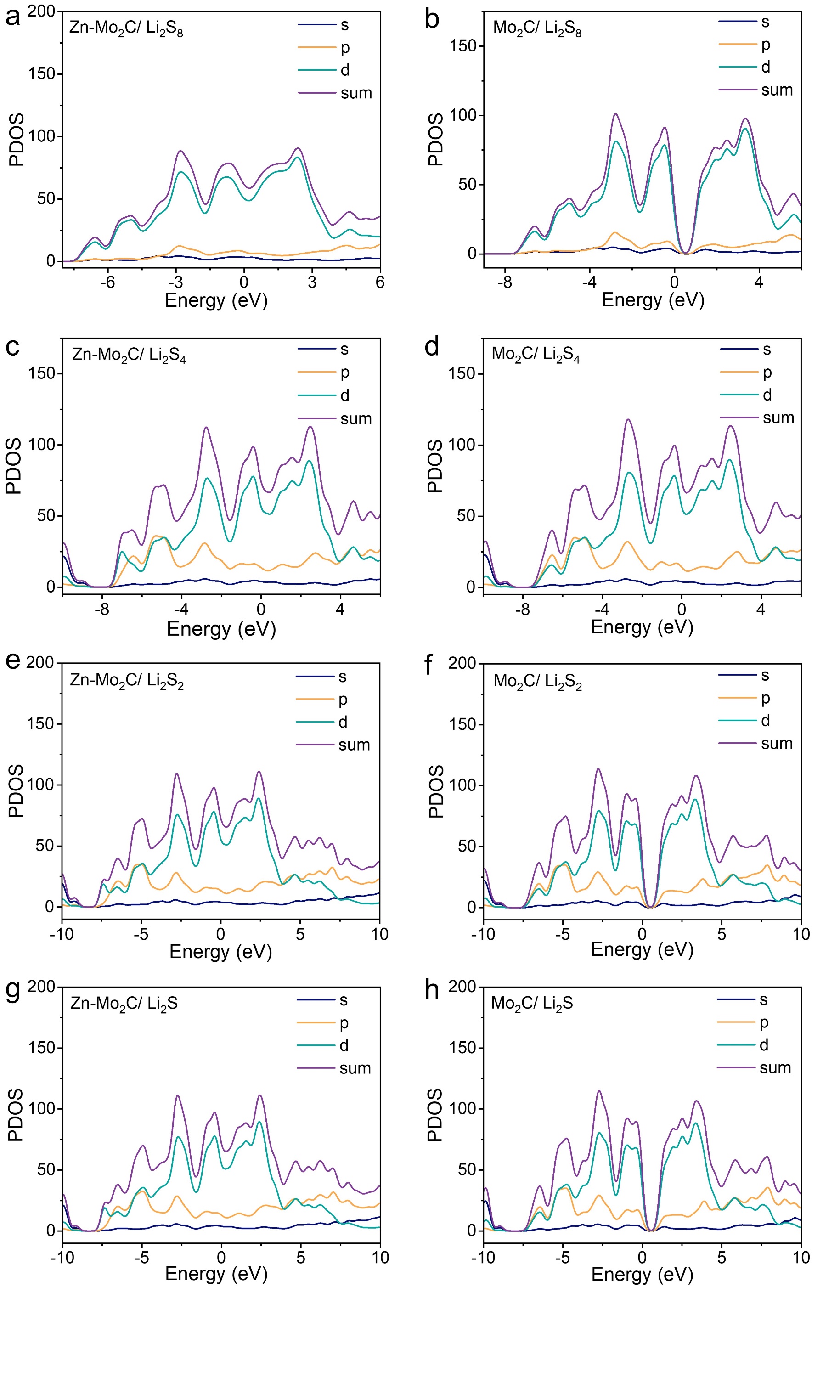


**Figure S25.** PDOS of Mo in (a) Zn-Mo_2_C/Li_2_S_8_, (b) Mo_2_C/Li_2_S_8_, (c) Zn-Mo_2_C/Li_2_S_4_, (d) Mo_2_C/Li_2_S_4_, (e) Zn-Mo_2_C/Li_2_S_2_, (f) Mo_2_C/Li_2_S_2_, (g) Zn-Mo_2_C/Li_2_S, (h) Mo_2_C/Li_2_S.

**Table S1.** Comparison of the lithium ion diffusion coefficient (*D*_Li_, cm^2^ s^–1^) paired with different separators: Zn-Mo_2_C and C-Mo_2_C.

| **Sample** | **Peak A** | **Peak B** | **Peak C** |
| --- | --- | --- | --- |
| Zn-Mo_2_C | 3.05 × 10^–7^ | 4.70 × 10^–8^ | 3.72 × 10^–7^ |
| C-Mo_2_C | 1.85 × 10^–7^ | 3.61 × 10^–8^ | 1.85 × 10^–7^ |

**Table S2.** Performance comparison of the Zn-Mo_2_C battery with the recently reported catalytic materials.

| **Samples** | **S loading**  **(mg cm**⁻**^2^)** | **Rate performance**  **(mAh g**⁻**^1^/C)** | **Reversible capacity**  **(mAh g**⁻**^1^/Cycles/C)** | **Decay rate (%)** | **Ref.** |
| --- | --- | --- | --- | --- | --- |
| **Zn-Mo_2_C** | **2.0** | **1300/0.2** | **849/500/2** | **0.025** | **This work** |
|  |  | **740/5** | **585/1000/5** | **0.021** |  |
| Co_7_Mo_3_B | 1.5 | 660.7/5 | 488.6/1000/5 | 0.026 | 2 |
| SP-Fe_3_O_4_-C | 1.2 | 1303.4/0.2 | 652.9/1200/1 | 0.027 | 3 |
|  |  | 691.8/5 |  |  |  |
| TiN−VN@CNFs | 1.2 | 1385/0.2 | 576/600/2 | 0.051 | 4 |
|  |  | 650/5 |  |  |  |
| MoS_2_−MoN/S | 1.2 | 778/2 | 459/1000/2 | 0.041 | 5 |
| S-MF@CNT | 1.5-2.0 | 1188/0.2 | 524.6/800/2 | 0.047 | 6 |
|  |  | 738/3 |  |  |  |
| S@CoSA-N_3_PS | 1.0 | 834/5 | 539/300/5 | 0.118 | 7 |
| Co-MoS_2_-G | 1.0 | 1220/0.3 | —/1000/1 | 0.029 | 8 |
| Co_3_O_4_/CoP | 1.4 | 782/1 | 665/500/1 | 0.03 | 9 |
| Zn_0.12_MoS_2_-CNFs | 1.5-2.0 | 1325/0.1 | 604/700/1 | 0.045 | 10 |
|  |  | 698/3 |  |  |  |
| CoNiO_2_/Co_4_N | 1.0 | 1198/0.2 | —/100/2 | 0.128 | 11 |
|  |  | 688/4 |  |  |  |

**Table S3.** Comparison of the S loading, S content, and specific capacity as well as the areal capacity of our work with recently reported works (sulfur loading > 3.0 mg cm⁻^2^).

| **Samples** | **S loadings**  **(mg cm**⁻**^2^)** | **S content**  **(wt.%)** | **Specific capacity**  **(mAh g**⁻**^1^)** | **Areal capacity**  **(mAh cm**⁻**^2^)** | **Ref.** |
| --- | --- | --- | --- | --- | --- |
| **Zn-Mo_2_C** | **6.0** | **79.6** | [**918.3@0.2**](mailto:1218@0.2) **C** | **5.5@0.2 C** | **This work** |
|  | **8.0** | **79.6** | **868.6@0.2 C** | **7.0@0.2 C** |  |
| SP-Fe_3_O_4_-C | 8.2 | 75 | — | 6.5@0.1 C | 3 |
| p-Co_3_O_4_/n-TiO_2_-HPs | 4.4 | 60 | — | 4.45@0.1 C | 12 |
| np-ANF | 5.8 | 66.7 | [1018@0.1](mailto:1018@0.1) C | 5.9[@0.1](mailto:1018@0.1) C | 13 |
| STMn_0.3_ | 3.0 | 70 | 730@0.2 C | — | 14 |
| MTQ@3DG | 4.9 | 76.2 | 1101.2@0.1 C | 5.4@0.1 C | 15 |
| MoO_2_−Mo_2_N−SP | 4.0 | 73 | 797@0.2 C | — | 16 |
| C_2_N@NbSe_2_ | 5.6 | 70.3 | — | 4.56@0.1 C | 17 |
| Ni@C/graphene | 4.0 | 70 | — | 3.8@0.5 C | 18 |
| Mo_2_C/CHS | 5.0 | 80 | 1031@0.1 C | — | 19 |
| Co_7_Fe_3_@  PGC−CNT | 6.7 | 70 | — | 6.1@0.1 C | 20 |
| TiO_2_-TiN | 3.0 | 73 | 610.8@0.3 C | — | 21 |
| CoFe-MCS | 7.7 | 78.2 | — | 6.0@0.1 C | 22 |
| Fe_3_C/NC | 5.0 | — | — | 3.6@0.2 C | 23 |
| ZnS/SnS_2_@NC | 5.9 | 78 | 642@0.5 C | 4.77@0.5 C | 24 |
| Fe-N_5_-C | 8.2 | 74 | — | 6.0@0.1 C | 25 |
| Fe-CoNC | 8.0 | 84 | — | 4.9@0.2 C | 26 |

# References

1. F. Ma, Y. Y. Wan, X. M. Wang, X. C. Wang, J. S. Liang, Z. P. Miao, T. Y. Wang, C. Ma, G. Lu, J. T. Han, Y. H. Huang, Q. Li, *ACS Nano* **2020**, *14*, 10115.
2. T. Feng, T. Zhao, N. X. Zhang, Y. Z. Duan, L. Li, F. Wu, R. J. Chen, *Adv. Funct. Mater.* **2022**, *32*, 2202766.
3. H. Zhang, M. T. Zhang, R. Y. Liu, T. F. He, L. X. Xiang, X. R. Wu, Z. H. Piao, Y. Y. Jia, C. Y. Zhang, H. Li, F. G. Xu, G. M. Zhou, Y. Y. Mai, *Nat. Commun.* **2024**, *15*, 1.
4. Y. Yao, H. Y. Wang, H. Yang, S. F. Zeng, R. Xu, F. F. Liu, P. C. Shi, Y. Z. Feng, K. Wang, W. J. Yang, X. J. Wu, W. Luo, Y. Yu, *Adv. Mater.* **2020**, *32*, 1905658.
5. S. Z. Wang, S. P. Feng, J. W. Liang, Q. M. Su, F. P. Zhao, H. J. Song, M. H. Zheng, Q. Sun, Z. X. Song, X. H. Jia, J. Yang, Y. Li, J. X. Liao, R. Y. Li, X. L. Sun, *Adv. Energy Mater.* **2021**, *11*, 2003314.
6. R. H. Gao, M. T. Zhang, Z. Y. Han, X. Xiao, X. R. Wu, Z. H. Piao, Z. J. Lao, L. Nie, S. G. Wang, G. M. Zhou, *Adv. Mater.* **2024**, *36*, 2303610.
7. T. T. Sun, F. D. Huang, J. L. Liu, H. Yu, X. Y. Feng, X. F. Feng, Y. Yang, H. B. Shu, F. Q. Zhang, *Adv. Funct. Mater.* **2023**, *33*, 2306049.
8. H. Lin, S. Zhang, T. Zhang, H. Ye, Q. Yao, G. W. Zheng, J. Y. Lee, *Adv. Energy Mater.* **2019**, *9*, 1902096.
9. D. R. Wang, D. Luo, Y. G. Zhang, Y. Zhao, G. F. Zhou, L. L. Shui, Z. W. Chen, X. Wang, *Nano Energy* **2021**, *81*, 105602.
10. M. J. Jin, G. W. Sun, Y. T. Wang, J. S. Yuan, H. X. Zhao, G. Wang, J. Y. Zhou, E. R. Xie, X. J. Pan, *ACS Nano* **2024**, *18*, 2017.
11. J. Pu, W. B. Gong, Z. X. Shen, L. T. Wang, Y. G. Yao, G. Hong, *Adv. Sci.* **2022**, 9, 2104375.
12. H. T. Li, C. Chen, Y. Y. Yan, T. R. Yan, C. Cheng, D. Sun, L. Zhang, *Adv. Mater.* **2021**, *33*, 2105067.
13. M. Q. Wang, A. E. Emre, J. Y. Kim, Y. T. Huang, L. Liu, V. Cecen, Y. D. Huang, N. A. Kotov, *Nat. Commun.* **2022**, *13*, 278.
14. W. S. Hou, P. L. Feng, X. Guo, Z. H. Wang, Z. Bai, Y. Bai, G. X. Wang, K. N. Sun, *Adv. Mater.* **2022**, *34*, 2202222.
15. B. Yu, A. J. Huang, K. Srinivas, X. J. Zhang, F. Ma, X. Q. Wang, D. J. Chen, B. Wang, W. L. Zhang, Z. G. Wang, J. R. He, Y. F. Chen, *ACS Nano* **2021**, *15*, 13279.
16. J. L. Yang, S. X. Zhao, Y. M. Lu, X. T. Zeng, W. Lv, G. Z. Cao, *Nano Energy* **2020**, *68*, 104356.
17. D. W. Yang, Z. F. Liang, C. Q. Zhang, J. J. Biendicho, M. Botifoll, M. C. Spadaro, Q. L. Chen, M. Y. Li, A. Ramon, A. O. Moghaddam, J. Llorca, J. A. Wang, J. R. Morante, J. Arbiol, S. L. Chou, A. Cabot, *Adv. Energy Mater.* **2021**, *11*, 2101250.
18. Z. Yu, B. L. Wang, X. B. Liao, K. N. Zhao, Z. F. Yang, F. J. Xia, C. L. Sun, Z. Wang, C. Y. Fan, J. P. Zhang, Y. G. Wang, *Adv. Energy Mater.* **2020**, *10*, 2000907.
19. J. Qian, Y. Xing, Y. Yang, Y. Li, K. X. Yu, W. L. Li, T. Zhao, Y. S. Ye, L. Li, F. Wu, R. J. Chen, *Adv. Mater.* **2021**, *33*, 2100810.
20. P. Zeng, C. Liu, X. F. Zhao, C. Yuan, Y. G. Chen, H. P. Lin, L. Zhang, *ACS Nano* **2020**, *14*, 11558.
21. P. Xue, K. P. Zhu, W. B. Gong, J. Pu, X. Y. Li, C. Guo, L. Y. Wu, R. Wang, H. P. Li, J. Y. Sun, G. Hong, Q. Zhang, Y. G. Yao, *Adv. Energy Mater.* **2022**, *12*, 2200308.
22. Z. X. Shi, Z. T. Sun, J. S. Cai, Z. D. Fan, J. Jin, M. L. Wang, J. Y. Sun, *Adv. Funct. Mater.* **2021**, *31*, 2006798.
23. C. Y. Zhou, X. C. Li, H. L. Jiang, Y. Ding, G. H. He, J. Guo, Z. Chu, G. H. Yu, *Adv. Funct. Mater.* **2021**, *31*, 2011249.
24. B. Yan, Y. Li, L. Gao, H. C. Tao, L. L. Zhang, S. K. Zhong, X. F. Li, X. L. Yang, *Small* **2022**, *18*, 2107727.
25. Y. G. Zhang, J. B. Liu, J. Y. Wang, Y. Zhao, D. Luo, A. P. Yu, X. Wang, Z. W. Chen, *Angew. Chem., Int. Ed.* **2021**, *60*, 26622.
26. C. X. Dong, C. Zhou, M. W. Wu, Y. K. Yu, K. S. Yu, K. J. Yan, C. L. Shen, J. P. Gu, M. Y. Yan, C. L. Sun, L. Q. Mai, X. Xu, *Adv. Energy Mater.* **2023**, *13*, 2301505.
